# Low-Carbohydrate Diet Macronutrient Quality and Weight Change

**DOI:** 10.1001/jamanetworkopen.2023.49552

**Published:** 2023-12-27

**Authors:** Binkai Liu, Yang Hu, Sharan K. Rai, Molin Wang, Frank B. Hu, Qi Sun

**Affiliations:** 1 Department of Nutrition, Harvard T.H. Chan School of Public Health, Boston, Massachusetts; 2 Department of Epidemiology, Harvard T.H. Chan School of Public Health, Boston, Massachusetts; 3 Channing Division of Network Medicine, Department of Medicine, Brigham and Women’s Hospital, Boston, Massachusetts; 4 Department of Biostatistics, Harvard T.H. Chan School of Public Health, Boston, Massachusetts

## Abstract

**Importance:**

The associations of low-carbohydrate diets (LCDs) with long-term weight management remains unclear, and the source and quality of macronutrients within LCDs are less explored.

**Objectives:**

To prospectively examine associations between changes in LCD indices and weight change among US adults.

**Design, Setting, and Participants:**

This prospective cohort study included initially healthy participants at baseline from the Nurses’ Health Study (NHS; 1986-2010), Nurses’ Health Study II (NHSII; 1991-2015), and Health Professionals Follow-up Study (HPFS; 1986-2018). Data analysis was performed between November 2022 and April 2023.

**Exposures:**

Five LCD indices were examined: (1) a total LCD (TLCD) emphasizing overall lower carbohydrate intake; (2) an animal-based LCD (ALCD) that emphasized animal-sourced protein and fat; (3) a vegetable-based LCD (VLCD) that emphasized plant-sourced protein and fat; (4) a healthy LCD (HLCD) emphasizing less refined carbohydrates, more plant protein, and healthy fat; and (5) an unhealthy LCD (ULCD) emphasizing less healthful carbohydrates, more animal protein, and unhealthy fat.

**Main Outcomes and Measures:**

The outcome of interest was 4-year changes in self-reported body weight.

**Results:**

A total of 123 332 participants (mean [SD] age, 45.0 [9.7] years; 103 320 [83.8%] female) were included in this study. The median carbohydrate intake (as a percentage of energy) of the highest quintiles of TLCD score at baseline ranged from 38.3% in HPFS to 40.9% in NHSII. Mean weight gain over 4-year intervals among participants varied from 0.8 kg in the HPFS to 1.8 kg in the NHSII. After adjusting for demographics and baseline and concomitant changes of selected lifestyle factors, each 1-SD increase in TLCD score was associated with 0.06 (95% CI, 0.04-0.08) kg more weight gain over the 4-year periods. Similarly, participants gained 0.13 (95% CI, 0.11 to 0.14) kg per each 1-SD increase in ALCD score and 0.39 (95% CI, 0.37 to 0.40) kg per each 1-SD change in ULCD score. In contrast, each 1-SD increase in VLCD score was associated with 0.03 (95% CI, 0.01 to 0.04) kg less weight gain, and each 1-SD increase in HLCD score was associated with 0.36 (95% CI, 0.35 to 0.38) kg less weight gain. The associations were more pronounced among obese individuals (per 1-SD increase in HLCD score: BMI ≥30, 0.88 [95% CI, 0.80, 0.97] kg less weight gain; BMI <25, 0.23 [95% CI, 0.20, 0.26] kg less weight gain; *P* for interaction < .001).

**Conclusions and Relevance:**

These findings suggest that the quality of LCDs may play a critical role in modulating long-term weight change. Only LCDs that emphasized high-quality protein, fat, and carbohydrates from whole grains and other plant-based foods were associated with less weight gain.

## Key Points

**Question:**

Are low-carbohydrate diets (LCDs) associated with long-term weight change, and does the source and quality of macronutrients within LCDs influence these associations?

**Findings:**

In this cohort study using data from 3 large prospective cohort studies among 123 332 individuals, LCDs that emphasized high-quality proteins, fats and carbohydrates from whole grains and other healthy plant-based foods were significantly associated with slower weight gain in the long term. In contrast, LCDs emphasizing animal-sourced proteins and fats or refined carbohydrates were associated with faster weight gain.

**Meaning:**

These findings underscore the importance of diet quality within LCD patterns for long-term weight management.

## Teaser

This cohort study examines associations of 5 low-carbohydrate diets including macronutrients of differing source and quality with weight change among US adults.

## Introduction

Low-carbohydrate diets (LCDs) have gained considerable attention, as they hold the promise for promoting weight loss and improving metabolic health.^1^ In clinical trials, LCDs, such as ketogenic diets with only 5% to 10% of energy from carbohydrates, have led to favorable short-term weight changes.^2,3^ However, whether these diets lead to long-term, favorable weight maintenance remains unknown. In addition, continued adherence to diets extremely low in carbohydrates may be unsustainable.^4^

Moreover, individual food groups with different qualities are associated with differential health consequences, including weight management. Whole grains, fruits, and nonstarchy vegetables are associated with less weight gain^5,6^; conversely, refined starches, sugar-sweetened beverages, and red and processed meat are detrimental to weight management and overall health.^6,7^ Few studies have considered the role of food group quality in the associations between LCDs and weight outcomes in free-living individuals with a range of carbohydrate intake typical for the general US population,^8^ who on average consume 50% energy as carbohydrates.^9^

To better understand the role of macronutrient quality in weight outcomes, we defined and evaluated 5 LCD indices in association with weight change with differential emphases on the quality of macronutrients. We prospectively examined the associations between changes in the LCD indices and weight change over 20 years of follow-up in US men and women.

## Methods

This cohort study was approved by the Human Research Committees of Brigham and Women’s Hospital and the Harvard T.H. Chan School of Public Health. Participants provided written informed consent by completing and returning study questionnaires. This study followed the Strengthening the Reporting of Observational Studies in Epidemiology (STROBE) reporting guideline.

### Study Population

We used data from 3 large ongoing prospective US cohorts: the Nurses’ Health Study (NHS), with 121 700 female nurses aged 30 to 55 years enrolled in 1976^10^; the Nurses’ Health Study II (NHSII) of 116 340 female nurses aged 25 to 42 years recruited in 1989^11^; and the Health Professionals Follow-up Study (HPFS), initiated in 1986 and enrolling 51 529 male health professionals aged 40 to 75 years.^12^ Lifestyle factors and medical history of the participants were assessed by biennial questionnaires.

In the current analysis, follow-up began in 1986 for NHS and HPFS and 1991 for NHSII, when diet was first comprehensively assessed. At baseline, we excluded participants with self-reported diabetes, cardiovascular disease, cancer (except nonmelanoma skin cancer), respiratory diseases, neurodegenerative disorders, gastric conditions, chronic kidney disease, or systemic lupus erythematosus, since these conditions may lead to substantial weight changes (eMethods in Supplement 1).^13^ Additional baseline exclusions were extreme energy intake (<600 or >3500 kcal/d for women; <800 or >4200 kcal/d for men) and missing data on baseline LCD score or weight. We also excluded participants older than 65 years at baseline, since subsequent weight changes are more likely to reflect a loss of lean muscle mass. During follow-up, we censored individuals when they reported a diabetes diagnosis and 6 years prior to diagnoses of cancer, respiratory diseases, neurodegenerative disorders, gastric conditions, chronic kidney disease, or lupus. We continued to censor individuals older than 65 years and those with missing data on LCD score change and weight change over the follow-up. Lastly, we excluded pregnant individuals for one 4-year cycle. After exclusions, the final baseline sample included 47 458 female nurses from the NHS, 55 862 female nurses from the NHSII, and 20 012 male health professionals from the HPFS. Self-reported race and ethnicity were assessed as basic demographic variables to examine their associations with LCD and weight change. We categorized race and ethnicity into the following groups: African American, Asian, Hispanic and other (ie, individual reported having Hispanic or other ancestry) White, or missing.

### Assessment of LCD Scores

Diet was assessed every 4 years using a validated semiquantitative food frequency questionnaire (FFQ). The validity and reproducibility of measurements on macronutrient intake levels have been reported in detail previously.^14,15,16,17^ We derived 5 LCD indices that emphasized different compositions and quality of macronutrients: (1) a total LCD (TLCD) emphasizing overall lower carbohydrate intake; (2) an animal-based LCD (ALCD) further emphasizing animal sources of protein and fat; (3) a vegetable-based LCD (VLCD) further emphasizing plant sources of protein and fat; (4) a healthy LCD (HLCD) emphasizing less refined carbohydrates, more plant protein, and healthy fat; and (5) an unhealthy LCD (ULCD) emphasizing less carbohydrates from healthful sources, such as whole grains, more animal protein, and unhealthy fat.^18^ Detailed computation methods of these scores can be found in the eMethods in Supplement 1. All scores ranged from 0 to 30, with a higher LCD score indicating greater adherence to the given LCD.

The primary exposure of interest was 4-year changes of the indices, which was calculated by subtracting the scores at the start of each 4-year interval from the scores at the end of the 4-year interval. For each 4-year cycle, the changes were divided into 5 quintiles (Q1 to Q5). The median quintile group (Q3) coincides with participants who had nearly no change in the scores. Accordingly, Q1 represents the largest decrease in a given score; Q2, a moderate decrease; Q3, no change in the score; Q4, a moderate increase; and Q5, the largest increase.

### Ascertainment of Weight Change and Covariates

The outcome of interest was 4-year weight change, calculated by subtracting weight at the start of each 4-year interval from weight at the end of the 4-year interval. Weight was self-reported at baseline and biennially thereafter. Weight has been previously validated in these cohorts, with correlation coefficients of 0.97 in NHS and HPFS and 0.84 in NHSII.^12,19^ Participants’ demographic and lifestyles factors, as well as the occurrence of new medical diagnoses, were collected biennially since baseline. Covariate assessment details can be found in the eMethods in Supplement 1.

### Statistical Analysis

Data analysis was performed between November 2022 and April 2023. We examined participants’ characteristics at baseline according to cohort and quintiles of each LCD index. We further examined the Spearman rank correlation coefficients *(r* values*)* between Alternative Healthy Eating Index–2010 (AHEI) and these LCD indices at baseline. The consumption levels of major food groups in the highest and lowest quintile of each score were presented. We used multivariable generalized linear regression models with an unstructured correlation matrix and robust variance to examine the associations of 4-year changes in the indices with concomitant 4-year weight changes using PROC GENMOD in SAS statistical software version 9.4 (SAS Institute) with a REPEATED statement. There were eight 4-year cycles for HPFS (1986-2018), and six 4-year cycles for both NHS (1986-2010) and NHSII (1991-2015). In each 4-year interval, associations of quintiles of change in each index, as well as per 1-SD increases in each index, were examined. The results were interpreted as mean weight change over 4-year intervals. *P* values for the linear trend across quintiles of indices changes were further calculated using median levels of each quintile as the continuous exposure in the model. All multivariable models adjusted for age, race and ethnicity, family history of diabetes, baseline hypertension, baseline hypercholesterolemia, baseline total caloric intake, baseline body mass index (BMI; calculated as weight in kilograms divided by height in meters squared), change in smoking status, baseline and change in physical activity, change in alcohol consumption, postmenopausal hormone use, and oral contraceptive use. All analyses were run in each cohort separately first, and analyses were repeated using pooled data from the 3 cohorts with further adjustment for data origins. Potential nonlinear associations were examined using restricted cubic spline regression using the SAS macro %GLMCURV9 with 3 knots, adjusting for the previously mentioned covariates.^20^ To minimize the influence of outliers, changes in weight and diet were truncated at the 0.5th and 99.5th percentiles. Furthermore, we conducted a sensitivity analysis using an independent correlation matrix.

We also examined potential association modification by age (<55 vs ≥55 years), BMI (<25, 25-30, ≥30), physical activity (below vs above median level, time-varying), and overall diet quality measured by AHEI (below vs above median level, time-varying). Stratified analyses were conducted in the combined data set by these modifiers. *P* values for interaction were calculated using the generalized score tests requested by the type 3 command in the model statement (equivalent to the likelihood ratio test). To further explore how the association between LCD change and weight change differed according to baseline BMI, results of the stratified analyses were presented using 3 different outcomes as the y-axis: (1) estimated 4-year weight change, (2) estimated 4-year changes in percentage of weight, and (3) estimated 4-year BMI change.

Analyses were conducted with the SAS for Unix and RStudio version 4.2.3 (R Project for Statistical Computing). Two-sided *P* < .05 was considered statistically significant.

## Results

### Baseline Characteristics

A total of 123 332 participants (mean [SD] age, 45.0 [9.7] years; 103 320 [83.8%] female) were included in this study. Participants included 1215 African American individuals (1.0%), 1328 Asian individuals (1.1%); 1277 Hispanic or other individuals (1.0%), 108 757 White individuals (88.2%), and 10 755 individuals missing race and ethnicity information (8.7%). Table 1 presents the baseline characteristics of study participants in extreme quintiles (Q1 and Q5) of baseline overall LCD scores (eTable 1 in Supplement 1). Comparing participants in Q5 vs Q1 TLCD score at baseline, the median (IQR) energy percentages from carbohydrates were 40.4% (37.1% to 42.8%) vs 57.2% (55.0% to 60.3%) in NHS, 40.9% (38.0% to 42.9%) vs 58.6% (56.3% to 62.0%) in NHSII, and 38.3% (34.9% to 41.1%) vs 56.8% (54.1% to 60.5%) in HPFS. Participants who were more adherent to an overall LCD at baseline were more likely to be current smokers, had lower physical activity levels, lower daily calorie intake, higher BMI, and were more likely to have a family history of diabetes (Table 1). The distributions of macronutrient intake and 5 LCD scores were similar in the 3 cohorts at baseline. Participant characteristics in the highest and lowest quintile of each LCD score in pooled data set are further shown in eTable 2 in Supplement 1. At baseline, HLCD and VLCD scores were positively correlated with AHEI, while ULCD, ALCD, and TLCD scores were negatively associated with AHEI, with *r* values ranging from −0.06 to −0.45 (eTable 3 in Supplement 1). The profile of major food group consumption for HLCD was composed of higher intakes of whole grains, nonstarchy vegetables, and fruits and lower intakes of dairy, red and processed meat, sugar-sweetened beverages, and sweets and desserts (eTable 4 in Supplement 1). Participants gained a mean (SD) of 1.3 (5.3) kg over each 4-year interval: participants in the NHS gained a mean (SD) 0.8 (4.9) kg, participants in the NHSII gained 1.8 (5.9) kg, and participants in the HPFS gained 0.5 (4.0) kg.

**Table 1.** Age-Standardized Characteristics of Participants at Baseline, Comparing Extreme Quintiles of Total Low-Carbohydrate Diet Score at Baseline^a^

Characteristic	Mean (SD)							
	NHS (n = 47 458)		NHSII (n = 55 862)^b^		HPFS (n = 20 012)		Pooled (n = 123 332)	
	Q1	Q5	Q1	Q5	Q1	Q5	Q1	Q5
No. of participants	9601	9717	12 024	10 342	3934	3995	25 559	26 258
Score								
TLCD	5 (3-7)	25 (23-27)	5 (2-7)	25 (24-27)	5 (3-7)	24 (23-27)	5 (3-7)	25 (23-27)
ALCD	5 (2-7)	25 (23-28)	4 (2-7)	26 (24-28)	5 (2-7)	25 (23-28)	4 (2-7)	25 (23-28)
VLCD	11 (8-15)	18 (14-21)	11 (7-15)	18 (15-22)	12 (9-15)	17 (14-21)	11 (8-15)	18 (14-21)
HLCD	13 (9-17)	16 (13-20)	13 (8-17)	17 (14-20)	13 (10-17)	15 (12-20)	13 (9-17)	16 (13-20)
ULCD	7 (4-11)	23 (20-26)	7 (4-11)	23 (20-26)	7 (4-11)	23 (20-26)	7 (4-11)	23 (20-26)
Age, y	52.9 (7.2)	51.5 (7.0)	36.7 (4.6)	37 (4.4)	50 (7.9)	51 (7.7)	44.8 (9.9)	44.5 (9.4)
Race and ethnicity, No. (%)								
African American	135 (1.4)	61 (0.6)	215 (1.8)	122 (1.2)	46 (1.2)	19 (0.5)	393 (1.5)	229 (0.9)
Asian	115 (1.2)	42 (0.4)	277 (2.3)	93 (0.9)	86 (2.2)	49 (1.2)	479 (1.9)	201 (0.8)
Hispanic or other	59 (0.6)	30 (0.3)	164 (1.4)	125 (1.2)	105 (2.7)	73 (1.8)	329 (1.3)	240 (0.9)
White	7594 (79.1)	7852 (80.8)	10987 (91.4)	9662 (93.4)	3533 (89.8)	3674 (92.0)	22060 (86.3)	23228 (88.5)
Missing	1698 (17.7)	1732 (17.8)	380 (3.2)	340 (3.3)	164 (4.2)	180 (4.5)	2298 (9.0)	2360 (9.0)
Current smoking, No. (%)	1605 (16.7)	2246 (23.1)	1239 (10.3)	1600 (15.5)	206 (5.2)	408 (10.2)	3052 (11.9)	4587 (17.5)
Alcohol intake, g/d	5 (9.3)	5.5 (8.3)	2.8 (5.5)	2.8 (5.1)	9.8 (14.1)	9.6 (11.3)	4.8 (9.2)	5.0 (8.1)
Physical activity, METs-h/wk	16 (21.8)	12.5 (19.2)	24.6 (33.3)	16.9 (22.7)	25.7 (29.5)	17.2 (21.1)	21.4 (29.1)	15.3 (21.2)
Total energy intake, kcal/d	1812 (530)	1684 (510)	1849 (556)	1640 (507)	2036 (614)	1935 (605)	1864 (561)	1709 (534)
Weight, kg	65.5 (12.3)	69.5 (14.0)	63.4 (13.1)	70.5 (16.9)	78.6 (10.6)	83.9 (12.3)	66.6 (13.5)	72.1 (16.0)
BMI	24.3 (4.2)	25.8 (4.9)	23.3 (4.5)	25.9 (5.9)	24.6 (2.8)	26.2 (3.5)	23.9 (4.2)	25.9 (5.2)
Alternative Healthy Eating Index–2010	52.9 (11.7)	51.9 (10.9)	49.3 (12.0)	47.2 (9.6)	54 (11.9)	51.2 (10.8)	51.5 (12.0)	49.6 (10.6)
Carbohydrates intake, median (IQR), % of energy								
Overall	57.2 (55.0-60.3)	40.4 (37.1-42.8)	58.6 (56.3-62.0)	40.9 (38.0-42.9)	56.8 (54.1-60.5)	38.3 (34.9-41.1)	57.8 (55.5-61.2)	40.6 (37.4-43.0)
High-quality^c^	17.0 (12.3-22.5)	11.5 (8.9-14.6)	14.6 (10.2-19.9)	9.9 (7.6-12.5)	17.1 (12.1-23.3)	10.3 (7.6-13.2)	15.9 (11.3-21.6)	10.6 (8.1-13.5)
Low-quality^c^	26.4 (22.2-31.0)	18.9 (15.6-22.2)	31.8 (26.7-37.9)	22.2 (19-25.2)	26.1 (21.4-31.3)	18.9 (15.6-22.1)	28.6 (23.7-34.3)	20.6 (17.1-23.9)
Protein intake, median (IQR), % of energy								
Overall	15.9 (14.4-17.3)	21.1 (19.5-22.9)	16.3 (14.6-17.8)	22.1 (20.5-24.0)	15.7 (14.1-17.2)	20.9 (19.4-22.8)	16.1 (14.5-17.6)	21.4 (19.8-23.3)
Animal-based	10.5 (9.1-11.9)	16.4 (14.8-18.4)	10.9 (9.2-12.4)	17.6 (15.9-19.6)	10.3 (8.8-11.7)	16.5 (14.9-18.4)	10.7 (9.1-12.1)	16.8 (15.2-18.9)
Plant-based	5.3 (4.6-6.0)	4.5 (4.0-5.1)	5.3 (4.5-6.3)	4.5 (3.9-5.0)	5.3 (4.6-6.2)	4.3 (3.8-5.0)	5.3 (4.5-6.2)	4.5 (4-5.1.0)
Fat intake, median (IQR), % of energy								
Overall	27.5 (24.7-29.9)	38.1 (35.6-40.8)	26.3 (23.4-28.6)	37.1 (34.8-39.8)	26.3 (23.0-29.0)	38.2 (35.7-41.0)	26.8 (23.8-29.2)	37.5 (35-40.3)
Animal-based	13.8 (11.6-16.0)	23.3 (20.7-26.2)	13 (10.8-15.0)	22.2 (19.9-24.7)	13.4 (10.7-15.7)	24.1 (21.3-27.2)	13.4 (11.1-15.5)	22.7 (20.1-25.6)
Plant-based	13.1 (10.6-15.7)	14.4 (11.6-17.5)	12.7 (10.6-15.1)	14.7 (12.2-17.4)	12.3 (9.9-14.8)	13.5 (10.9-16.7)	12.8 (10.5-15.3)	14.4 (11.8-17.4)
Baseline hypertension, No. (%)	2102 (21.9)	2252 (23.2)	655 (5.4)	792 (7.7)	632 (16.1)	692 (17.3)	3470 (13.6)	3963 (15.1)
Baseline hypercholesterolemia, No. (%)	1209 (12.6)	977 (10.1)	1693 (14.1)	1612 (15.6)	458 (11.7)	345 (8.6)	3375 (13.2)	3192 (12.2)
Family history of diabetes, No. (%)	2370 (24.7)	2625 (27.0)	1834 (15.3)	1932 (18.7)	761 (19.4)	898 (22.5)	5005 (19.6)	5875 (22.4)
Any use of postmenopausal hormone, No. (%)	2498 (26.0)	2588 (26.6)	408 (3.4)	319 (3.1)	NA	NA	NA	NA
Use of oral contraceptives, No. (%)	NA	NA	2143 (17.8)	1439 (13.9)	NA	NA	NA	NA

Abbreviations: ALCD, animal-based low-carbohydrate diet; BMI, body mass index (calculated as weight in kilograms divided by height in meters squared); HLCD, healthy low-carbohydrate diet; HPFS, Health Professionals Follow-up Study; NA, not applicable; NHS, Nurses’ Health Study; NHSII, Nurses’ Health Study II; Q1, quintile 1 (lowest score); Q5, quintile 5 (highest score); TLCD, total low-carbohydrate diet; ULCD, unhealthy low-carbohydrate diet; VLCD, plant-based low-carbohydrate diet.

^a^ Complete participant characteristics at baseline are included in eTable 1 in Supplement 1.

^b^ A total of 61 949 participants in NHSII were included for data analysis, but a significant number of them were pregnant at baseline and were included in later cycles.

^c^ High-quality carbohydrates include fruit carbohydrate (excluding juice sugar), vegetable carbohydrate (excluding potato), and sum of carbohydrate from whole grains. Low-quality carbohydrates include sum of carbohydrate from potato, added sugar in foods, and refined grains.

### LCD Scores and Weight Change

After adjusting for baseline and concomitant changes in lifestyle and demographic factors, compared with participants with no change (Q3) in TLCD score over 4-year intervals, those who had the largest increase (Q5) in TLCD score did not have significant weight change (0.03 [95% CI, −0.02 to 0.07] kg), while those who had the largest decrease (Q1) of TLCD score had significantly less weight gain (−0.20 [95% CI, −0.25 to −0.15] kg) (Table 2). Similarly, participants following a VLCD with Q5 change, compared with those with stable Q3 adherence, experienced 0.21 (95% CI, 0.17 to 0.26) kg less weight gain, and those with Q1 change experienced 0.17 (95% CI, 0.12 to 0.22) kg less weight gain. Adhering to an ALCD was more likely to be associated with more weight gain over time. Each 1-SD increase of ALCD score was associated with 0.13 (95% CI, 0.11 to 0.14) kg more weight gain over 4-year intervals. Moreover divergent associations were observed for ULCD and HLCD scores, where a 1-SD increase of HLCD score was associated with a weight change of −0.36 (95% CI, −0.38 to −0.35) kg and ULCD was associated with a weight change of 0.39 kg (95% CI, 0.37 to 0.40) kg over a 4-year interval. Compared with participants with stable HLCD (Q3) over 4-year intervals, those who were in Q5 of HLCD change had 0.64 (95% CI, 0.60 to 0.69) kg less weight gain, and those in Q1 of HLCD change had 0.32 (95% CI, 0.28 to 0.37) kg more weight gain. In contrast, the same comparison for ULCD was associated with 0.42 (95% CI, 0.38 to 0.47) kg more weight gain for Q5 vs Q3, and 0.60 (95% CI, 0.55 to 0.65) kg less weight gain for Q1 vs Q3 over time. Overall, there were significant linear trends across quintiles of changes in TLCD, ALCD, HLCD, and ULCD (Table 2). In the sensitivity analysis using an independent instead of an unstructured correlation matrix, the results remained unchanged (eTable 5 in Supplement 1).

**Table 2.** Weight Changes Over 4-Year Periods According to 4-Year Change in LCD Scores

Measure	Q1	Q2	Q3	Q4	Q5	*P* for trend^a^	Change per 1-SD
**TLCD Score**							
NHS							
Median (IQR) index change	−9 (−12 to −7)	−3 (−4 to −2)	0 (−1 to 1)	3 (2 to 4)	9 (7 to 11)	NA	NA
Weight change, kg							
Overall, mean (SD)	0.63 (5.16)	0.79 (4.75)	0.87 (4.71)	0.96 (4.68)	0.92 (4.95)	NA	NA
Age adjusted, mean (95% CI)	−0.17 (−0.24 to −0.10)	−0.06 (−0.12 to 0.01)	0 [Reference]	0.09 (0.02 to 0.15)	0.05 (−0.02 to 0.12)	<.0001	0.08 (0.05 to 0.10)
Multivariable adjusted, mean (95% CI)^b^	−0.21 (−0.28 to −0.14)	−0.08 (−0.15 to −0.02)	0 [Reference]	0.10 (0.03 to 0.16)	0.06 (−0.01 to 0.12)	<.0001	0.10 (0.07 to 0.12)
NHSII							
Median (IQR) index change	−9 (−12 to −8)	−4 (−5 to −2)	0 (−1 to 1)	3 (2 to 5)	9 (7 to 13)	NA	NA
Weight change, kg							
Overall, mean (SD)	1.67 (6.23)	1.89 (5.72)	1.86 (5.62)	1.96 (5.70)	1.72 (6.13)	NA	NA
Age adjusted, mean (95% CI)	−0.14 (−0.22 to −0.07)	−0.00 (−0.07 to 0.07)	0 [Reference]	0.05 (−0.02 to 0.12)	−0.10 (−0.17 to −0.02)	.23	0.01 (−0.01 to 0.04)
Multivariable adjusted, mean (95% CI)^b^	−0.19 (−0.26 to −0.11)	−0.01 (−0.08 to 0.06)	0 [Reference]	0.05 (−0.02 to 0.12)	−0.10 (−0.18 to −0.03)	.03	0.03 (−0.00 to 0.05)
**HPFS**							
Median (IQR) index change	−8 (−11 to −7)	−3 (−4 to −2)	0 (−1 to 1)	3 (2 to 4)	8 (7 to 11)	NA	NA
Weight change, kg							
Overall, mean (SD)	0.23 (4.21)	0.47 (3.87)	0.61 (3.81)	0.68 (3.91)	0.60 (4.17)	NA	NA
Age adjusted, mean (95% CI)	−0.32 (−0.41 to −0.22)	−0.10 (−0.18 to −0.01)	0 [Reference]	0.08 (−0.01 to 0.16)	0.03 (−0.06 to 0.12)	<.0001	0.13 (0.10 to 0.17)
Multivariable adjusted, mean (95% CI)^b^	−0.34 (−0.43 to −0.25)	−0.10 (−0.19 to −0.02)	0 [Reference]	0.09 (0.01 to 0.18)	0.05 (−0.04 to 0.14)	<.0001	0.15 (0.11 to 0.18)
Pooled							
Median (IQR) index change	−9 (−12 to −7)	−3 (−5 to −2)	0 (−1 to 1)	3 (2 to 4)	9 (7 to 12)	NA	NA
Weight change, kg							
Overall, mean (SD)	1.07 (5.61)	1.28 (5.18)	1.29 (5.06)	1.40 (5.14)	1.25 (5.46)	NA	NA
Age adjusted, mean (95% CI)	−0.17 (−0.21 to −0.12)	−0.03 (−0.07 to 0.02)	0 [Reference]	0.08 (0.03 to 0.12)	−0.02 (−0.06 to 0.03)	<.0001	0.05 (0.04 to 0.07)
Multivariable adjusted, mean (95% CI)^b^	−0.20 (−0.25 to −0.15)	−0.04 (−0.09 to −0.00)	0 [Reference]	0.08 (0.03 to 0.12)	−0.03 (−0.07 to 0.02)	<.0001	0.06 (0.04 to 0.08)
**ALCD Score**							
NHS							
Median (IQR) index change	−10 (−13 to −8)	−4 (−5 to −2)	0 (−1 to 1)	4 (2 to 5)	10 (8 to 13)	NA	NA
Weight change, kg							
Overall, mean (SD)	0.62 (5.14)	0.84 (4.77)	0.84 (4.65)	0.98 (4.69)	0.89 (4.99)	NA	NA
Age adjusted, mean (95% CI)	−0.17 (−0.24 to −0.10)	−0.02 (−0.08 to 0.05)	0 [Reference]	0.13 (0.06 to 0.20)	0.06 (−0.01 to 0.13)	<.0001	0.09 (0.06 to 0.11)
Multivariable adjusted, mean (95% CI)^b^	−0.20 (−0.27 to −0.13)	−0.04 (−0.10 to 0.03)	0 [Reference]	0.12 (0.06 to 0.19)	0.07 (−0.00 to 0.14)	<.0001	0.10 (0.08 to 0.13)
NHSII							
Median (IQR) index change	−10 (−13 to −8)	−4 (−5 to −3)	0 (−1 to 1)	4 (3 to 5)	10 (8 to 13)	NA	NA
Weight change, kg							
Overall, mean (SD)	1.61 (6.20)	1.85 (5.77)	1.83 (5.59)	1.91 (5.67)	1.91 (6.10)	NA	NA
Age adjusted, mean (95% CI)	−0.20 (−0.27 to −0.12)	0.00 (−0.07 to 0.07)	0 [Reference]	0.08 (0.01 to 0.15)	0.14 (0.07 to 0.22)	<.0001	0.12 (0.10 to 0.15)
Multivariable adjusted, mean (95% CI)^b^	−0.23 (−0.31 to −0.16)	−0.01 (−0.08 to 0.06)	0 [Reference]	0.08 (0.01 to 0.15)	0.12 (0.05 to 0.20)	<.0001	0.13 (0.10 to 0.16)
HPFS							
Median (IQR) index change	−9 (−12 to −7)	−3 (−4 to −2)	0 (−1 to 1)	3 (2 to 4)	9 (7 to 12)	NA	NA
Weight change, kg							
Overall, mean (SD)	0.21 (4.18)	0.45 (3.85)	0.61 (3.89)	0.65 (3.87)	0.69 (4.15)	NA	NA
Age adjusted, mean (95% CI)	−0.36 (−0.45 to −0.27)	−0.12 (−0.21 to −0.04)	0 [Reference]	0.05 (−0.03 to 0.14)	0.14 (0.05 to 0.23)	<.0001	0.19 (0.16 to 0.22)
Multivariable adjusted, mean (95% CI)^b^	−0.37 (−0.46 to −0.28)	−0.13 (−0.22 to −0.04)	0 [Reference]	0.06 (−0.03 to 0.15)	0.14 (0.05 to 0.23)	<.0001	0.19 (0.16 to 0.23)
Pooled							
Median (IQR) index change	−10 (−13 to −8)	−4 (−5 to −2)	0 (−1 to 1)	4 (2 to 5)	10 (8 to 13)	NA	NA
Weight change, kg							
Overall, mean (SD)	1.04 (5.59)	1.26 (5.19)	1.27 (5.04)	1.37 (5.11)	1.36 (5.48)	NA	NA
Age adjusted, mean (95% CI)	−0.20 (−0.25 to −0.16)	−0.01 (−0.06 to 0.03)	0 [Reference]	0.10 (0.06 to 0.15)	0.11 (0.07 to 0.16)	<.0001	0.12 (0.10 to 0.13)
Multivariable adjusted, mean (95% CI)^b^	−0.23 (−0.28 to −0.18)	−0.03 (−0.08 to 0.01)	0 [Reference]	0.10 (0.05 to 0.14)	0.11 (0.06 to 0.16)	<.0001	0.13 (0.11 to 0.14)
**VLCD Score**							
NHS							
Median (IQR) index change	−7 (−9 to −6)	−3 (−4 to −2)	0 (−1 to 1)	3 (2 to 4)	7 (6 to 9)	NA	NA
Weight change, kg							
Overall, mean (SD)	0.57 (5.05)	0.78 (4.79)	0.93 (4.71)	0.94 (4.73)	0.94 (4.98)	NA	NA
Age adjusted, mean (95% CI)	−0.26 (−0.33 to −0.19)	−0.12 (−0.18 to −0.05)	0 [Reference]	0.03 (−0.03 to 0.10)	0.03 (−0.04 to 0.09)	<.0001	0.09 (0.07 to 0.12)
Multivariable adjusted, mean (95% CI)^b^	−0.29 (−0.35 to −0.22)	−0.12 (−0.19 to −0.06)	0 [Reference]	0.03 (−0.03 to 0.10)	0.02 (−0.04 to 0.09)	<.0001	0.10 (0.08 to 0.13)
NHSII							
Median (IQR) index change	−7 (−9 to −6)	−3 (−4 to −2)	0 (−1 to 1)	3 (2 to 4)	7 (6 to 10)	NA	NA
Weight change, kg							
Overall, mean (SD)	1.81 (6.16)	1.89 (5.72)	1.94 (5.63)	1.89 (5.68)	1.57 (6.17)	NA	NA
Age adjusted, mean (95% CI)	−0.05 (−0.12 to 0.02)	−0.02 (−0.09 to 0.05)	0 [Reference]	−0.07 (−0.14 to −0.00)	−0.35 (−0.42 to −0.28)	<.0001	−0.12 (−0.15 to −0.09)
Multivariable adjusted, mean (95% CI)^b^	−0.07 (−0.14 to −0.00)	−0.02 (−0.09 to 0.04)	0 [Reference]	−0.07 (−0.13 to 0.00)	−0.35 (−0.42 to −0.28)	<.0001	−0.11 (−0.14 to −0.08)
HPFS							
Median (IQR) index change	−7 (−9 to −5)	−3 (−4 to −2)	0 (−1 to 1)	3 (2 to 3)	7 (5 to 9)	NA	NA
Weight change, kg							
Overall, mean (SD)	0.32 (4.23)	0.53 (3.95)	0.70 (3.79)	0.54 (3.87)	0.46 (4.17)	NA	NA
Age adjusted, mean (95% CI)	−0.31 (−0.40 to −0.22)	−0.14 (−0.23 to −0.06)	0 [Reference]	−0.17 (−0.25 to −0.09)	−0.23 (−0.32 to −0.14)	.29	0.02 (−0.02 to 0.05)
Multivariable adjusted, mean (95% CI)^b^	−0.32 (−0.41 to −0.23)	−0.15 (−0.23 to −0.07)	0 [Reference]	−0.16 (−0.25 to −0.08)	−0.23 (−0.32 to −0.14)	.20	0.02 (−0.01 to 0.05)
Pooled							
Median (IQR) index change	−7 (−9 to −6)	−3 (−4 to −2)	0 (−1 to 1)	3 (2 to 4)	7 (6 to 9)	NA	NA
Weight change, kg							
Overall, mean (SD)	1.14 (5.57)	1.27 (5.19)	1.38 (5.08)	1.31 (5.10)	1.18 (5.51)	NA	NA
Age adjusted, mean (95% CI)	−0.15 (−0.20 to −0.11)	−0.07 (−0.11 to −0.03)	0 [Reference]	−0.05 (−0.09 to −0.01)	−0.19 (−0.23 to −0.15)	.30	−0.02 (−0.04 to −0.01)
Multivariable adjusted, mean (95% CI)^b^	−0.17 (−0.22 to −0.12)	−0.06 (−0.11 to −0.02)	0 [Reference]	−0.05 (−0.10 to −0.01)	−0.21 (−0.26 to −0.17)	.16	−0.03 (−0.04 to −0.01)
**HLCD Score**							
NHS							
Median (IQR) index change	−7 (−9 to −6)	−3 (−4 to −2)	0 (−1 to 1)	3 (2 to 4)	7 (6 to 9)	NA	NA
Weight change, kg							
Overall, mean (SD)	0.91 (4.98)	0.98 (0.74)	0.93 (4.75)	0.78 (4.80)	0.56 (5.00)	NA	NA
Age adjusted, mean (95% CI)	0.09 (0.02 to 0.15)	0.07 (0.01 to 0.14)	0 [Reference]	−0.15 (−0.21 to −0.08)	−0.38 (−0.44 to −0.31)	<.0001	−0.19 (−0.21 to −0.16)
Multivariable adjusted, mean (95% CI)^b^	0.06 (−0.00 to 0.13)	0.08 (0.01 to 0.14)	0 [Reference]	−0.13 (−0.20 to −0.07)	−0.37 (−0.44 to −0.31)	<.0001	−0.18 (−0.20 to −0.15)
NHSII							
Median (IQR) index change	−7 (−9 to −6)	−3 (−4 to −2)	0 (−1 to 1)	3 (2 to 4)	7 (6 to 10)	NA	NA
Weight change, kg							
Overall, mean (SD)	2.37 (6.08)	2.11 (5.65)	1.91 (5.66)	1.68 (5.68)	1.00 (6.23)	NA	NA
Age adjusted, mean (95% CI)	0.55 (0.48 to 0.62)	0.23 (0.16 to 0.30)	0 [Reference]	−0.24 (−0.31 to −0.18)	−0.86 (−0.93 to −0.79)	<.0001	−0.52 (−0.55 to −0.50)
Multivariable adjusted, mean (95% CI)^b^	0.52 (0.45 to 0.59)	0.22 (0.16 to 0.29)	0 [Reference]	−0.24 (−0.31 to −0.17)	−0.86 (−0.93 to −0.79)	<.0001	−0.51 (−0.54 to −0.49)
HPFS							
Median (IQR) index change	−7 (−9 to −5)	−3 (−4 to −2)	0 (−1 to 1)	3 (2 to 4)	7 (6 to 9)	NA	NA
Weight change, kg							
Overall, mean (SD)	0.65 (4.12)	0.71 (3.93)	0.62 (3.83)	0.44 (3.90)	0.15 (4.22)	NA	NA
Age adjusted, mean (95% CI)	0.10 (0.02 to 0.19)	0.11 (0.02 to 0.19)	0 [Reference]	−0.17 (−0.26 to −0.09)	−0.47 (−0.56 to −0.38)	<.0001	−0.22 (−0.26 to −0.19)
Multivariable adjusted, mean (95% CI)^b^	0.08 (−0.01 to 0.17)	0.10 (0.01 to 0.18)	0 [Reference]	−0.16 (−0.25 to −0.08)	−0.47 (−0.56 to −0.38)	<.0001	−0.21 (−0.25 to −0.18)
Pooled							
Median (IQR) index change	−7 (−9 to −6)	−3 (−4 to −2)	0 (−1 to 1)	3 (2 to 4)	7 (6 to 9)	NA	NA
Weight change, kg							
Overall, mean (SD)	1.59 (5.51)	1.48 (5.14)	1.35 (5.11)	1.14 (5.13)	0.71 (5.54)	NA	NA
Age adjusted, mean (95% CI)	0.33 (0.28 to 0.37)	0.15 (0.11 to 0.20)	0 [Reference]	−0.19 (−0.23 to −0.15)	−0.62 (−0.66 to −0.57)	<.0001	−0.36 (−0.37 to −0.34)
Multivariable adjusted, mean (95% CI)^b^	0.32 (0.28 to 0.37)	0.16 (0.12 to 0.20)	0 [Reference]	−0.19 (−0.24 to −0.15)	−0.64 (−0.69 to −0.60)	<.0001	−0.36 (−0.38 to −0.35)
**ULCD Score**							
NHS							
Median (IQR) index change	−8 (−11 to −6)	−3 (−4 to −2)	0 (−1 to 1)	3 (2 to 4)	8 (7 to 11)	NA	NA
Weight change, kg							
Overall, mean (SD)	0.32 (5.14)	0.79 (4.77)	0.89 (4.65)	1.09 (4.69)	1.09 (4.98)	NA	NA
Age adjusted, mean (95% CI)	−0.50 (−0.57 to −0.43)	−0.12 (−0.18 to −0.05)	0 [Reference]	0.21 (0.15 to 0.27)	0.27 (0.20 to 0.33)	<.0001	0.29 (0.27 to 0.32)
Multivariable adjusted, mean (95% CI)^b^	−0.54 (−0.61 to −0.47)	−0.14 (−0.21 to −0.08)	0 [Reference]	0.22 (0.16 to 0.28)	0.28 (0.22 to 0.35)	<.0001	0.32 (0.29 to 0.34)
NHSII							
Median (IQR) index change	−9 (−11 to −7)	−3 (−4 to −2)	0 (−1 to 1)	3 (2 to 4)	9 (7 to 11)	NA	NA
Weight change, kg							
Overall, mean (SD)	1.20 (6.29)	1.67 (5.64)	1.84 (5.58)	2.07 (5.68)	2.32 (6.15)	NA	NA
Age adjusted, mean (95% CI)	−0.62 (−0.69 to −0.54)	−0.15 (−0.22 to −0.08)	0 [Reference]	0.26 (0.19 to 0.33)	0.56 (0.49 to 0.63)	<.0001	0.43 (0.40 to 0.46)
Multivariable adjusted, mean (95% CI)^b^	−0.66 (−0.74 to −0.59)	−0.17 (−0.23 to −0.10)	0 [Reference]	0.25 (0.18 to 0.32)	0.53 (0.46 to 0.61)	<.0001	0.44 (0.41 to 0.47)
HPFS							
Median (IQR) index change	−7 (−10 to −6)	−3 (−4 to −2)	0 (−1 to 1)	3 (2 to 4)	7 (6 to 10)	NA	NA
Weight change, kg							
Overall, mean (SD)	0.02 (4.24)	0.47 (3.85)	0.57 (3.76)	0.74 (3.87)	0.85 (4.16)	NA	NA
Age adjusted, mean (95% CI)	−0.51 (−0.60 to −0.43)	−0.10 (−0.19 to −0.02)	0 [Reference]	0.18 (0.09 to 0.26)	0.35 (0.27 to 0.44)	<.0001	0.35 (0.31 to 0.38)
Multivariable adjusted, mean (95% CI)^b^	−0.54 (−0.63 to −0.46)	−0.12 (−0.21 to −0.04)	0 [Reference]	0.19 (0.11 to 0.27)	0.36 (0.28 to 0.45)	<.0001	0.36 (0.33 to 0.40)
Pooled							
Median (IQR) index change	−8 (−11 to −6)	−3 (−4 to −2)	0 (−1 to 1)	3 (2 to 4)	8 (7 to 11)	NA	NA
Weight change, kg							
Overall, mean (SD)	0.68 (5.60)	1.19 (5.15)	1.28 (5.02)	1.51 (5.13)	1.64 (5.51)	NA	NA
Age adjusted, mean (95% CI)	−0.56 (−0.60 to −0.51)	−0.12 (−0.16 to −0.07)	0 [Reference]	0.24 (0.20 to 0.28)	0.42 (0.37 to 0.46)	<.0001	0.37 (0.35 to 0.38)
Multivariable adjusted, mean (95% CI)^b^	−0.60 (−0.65 to −0.55)	−0.15 (−0.20 to −0.11)	0 [Reference]	0.23 (0.19 to 0.28)	0.42 (0.38 to 0.47)	<.0001	0.39 (0.37 to 0.40)

Abbreviations: ALCD, animal-based low-carbohydrate diet; HLCD, healthy low-carbohydrate diet; NA, not applicable; Q1, quintile 1 (large decrease); Q2, quintile 2 (moderate decrease); Q3, quintile 3 (no change); Q4, quintile 4 (moderate increase); Q5, quintile 5 (large increase); TLCD, total low-carbohydrate diet; ULCD, unhealthy low-carbohydrate diet; VLCD, vegetable-based low-carbohydrate diet.

^a^ *P* for trend values were calculated using median levels of each quintile as the continuous exposure in the model.

^b^ Multivariable adjusted models adjusted for age, race and ethnicity, family history of diabetes, baseline hypertension, baseline hypercholesterolemia, baseline total caloric intake, baseline body mass index, change in smoking status, baseline and change in physical activity, change in alcohol consumption, postmenopausal hormone use (women only), oral contraceptive use (NHSII only). Pooled results were further adjusted for cohort.

The results across the 3 cohorts were largely consistent, where increasing TLCD, ALCD, and ULCD scores were each associated with more weight gain, and increasing HLCD score was associated with less weight gain (Table 2). The results for VLCD were more heterogenous across the 3 cohorts: in NHSII, changing to more adherence to VLCD was significantly associated with less weight gain, while the association was less clear in NHS and HPFS.

**Figure 1.**
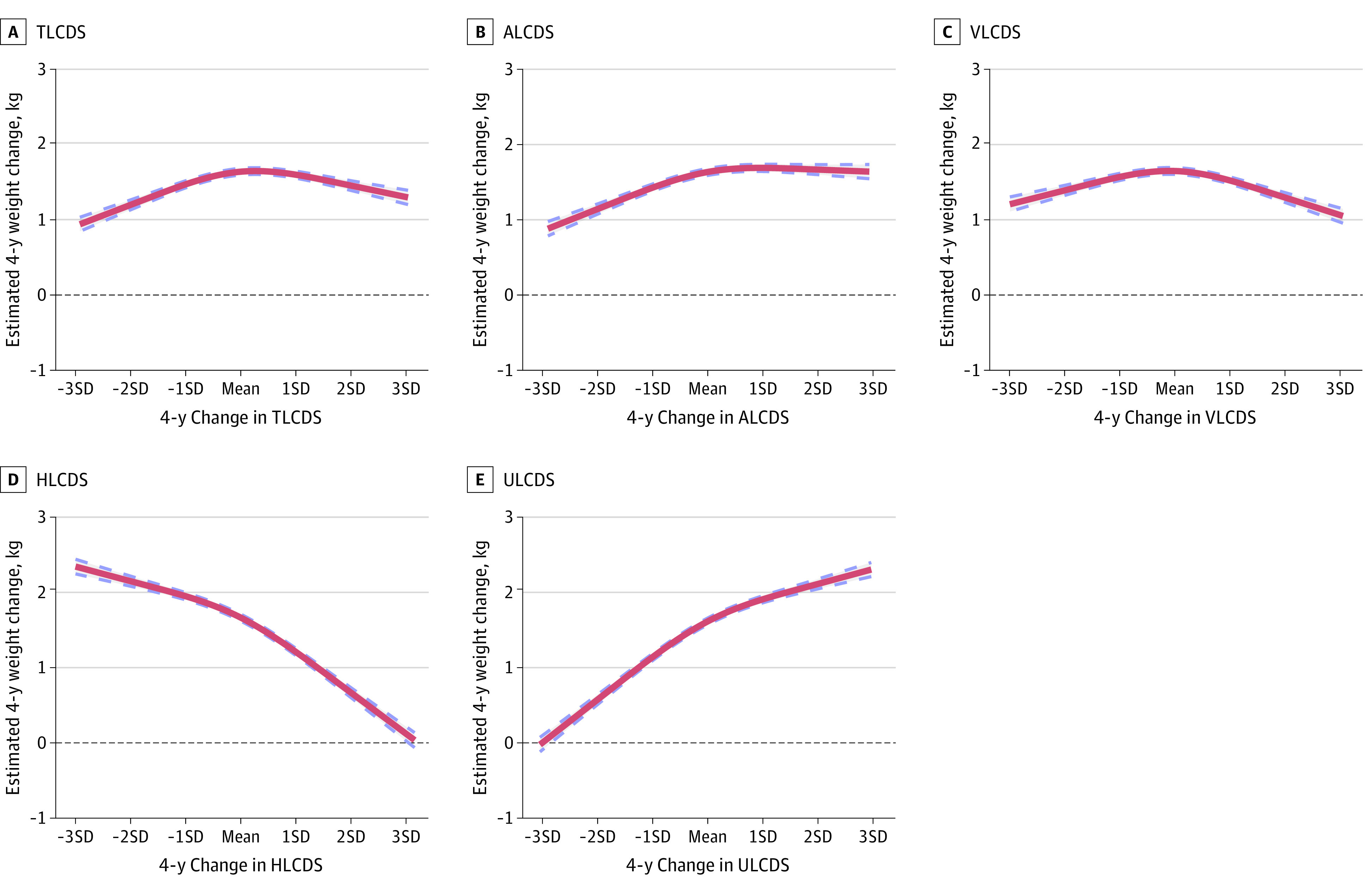
Adjusted Nonlinear Associations Between 4-Year Change in Low-Carbohydrate Diet (LCD) Scores and 4-Year Weight Changes *P* for curvature < .001 for all 5 associations. ALCDS indicates animal LCD score; HLCDS, healthy LCD score; TLCDS, total LCD score; ULCDS, unhealthy LCD score; VLCDS, vegetable low-carbohydrate diet score. Red line indicates mean; dashed lines, 95% CIs.

Restricted cubic spline regression showed a nonlinear dose-response association between 4-year changes in all 5 LCDs and weight change (*P* < .001 for curvature for all 5 associations) (Figure 1). All curves were above zero since the overall trend was for participants to gain weight over time. An inverted U-shape was observed for TLCD and VLCD. Although the associations were statistically significant, the nonlinear association for changes in HLCD and ULCD were much less apparent.

### Subgroup Analysis

**Figure 2.**
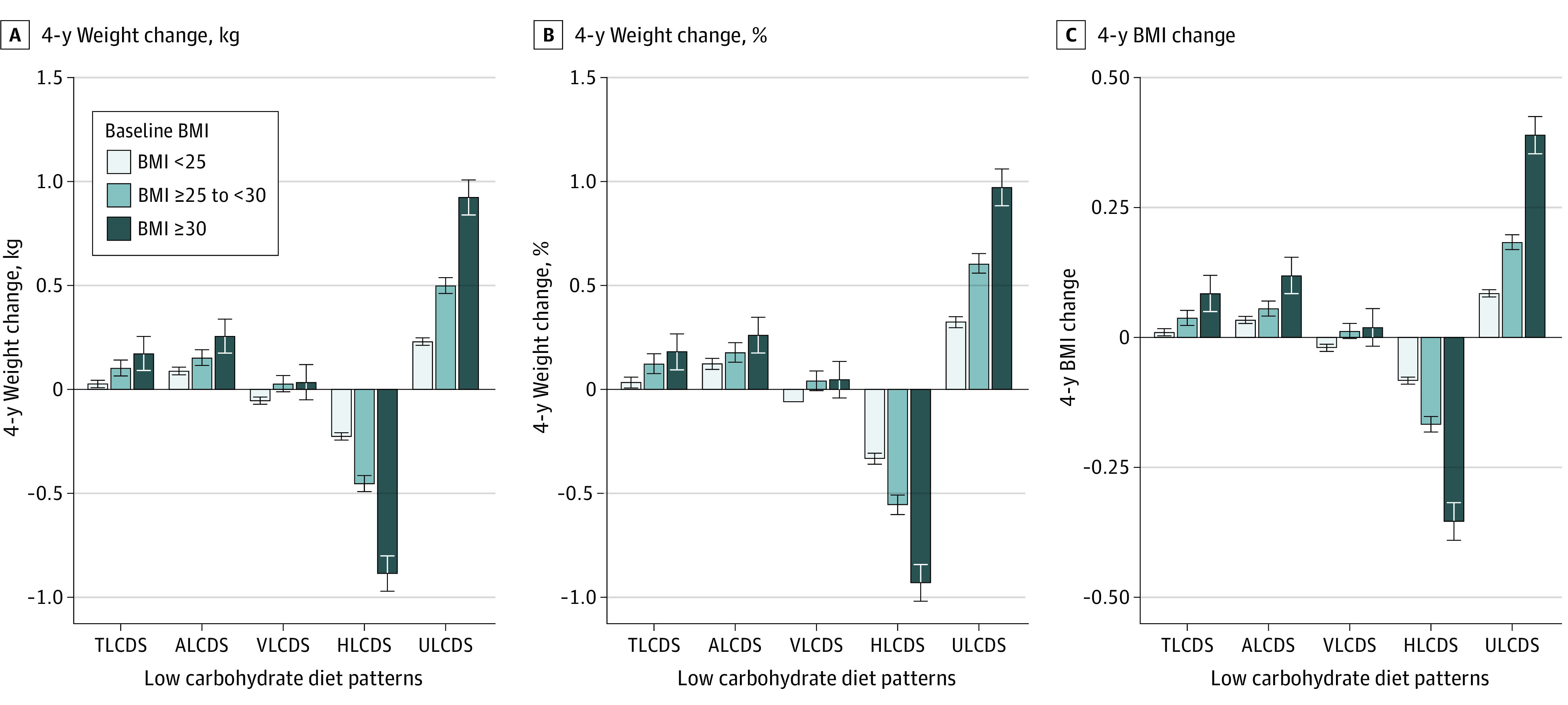
Association of Low-Carbohydrate Diet With 4-Year Weight Change Stratified by Baseline Body Mass Index (BMI) BMI is calculated as weight in kilograms divided by height in meters squared. Error bars indicate 95% CIs. ALCDS indicates animal LCD score; HLCDS, healthy LCD score; TLCDS, total LCD score; ULCDS, unhealthy LCD score; VLCDS, vegetable low-carbohydrate diet score.

In the stratified analysis, participants who were younger (<55 y), were less physically active, had overweight or obesity, and had lower overall diet quality (measured by AHEI) were more likely to experience less weight gain when adhering to HLCD, although the directionality of the associations was the same and significant in all subgroups (Table 3). In particular, among individuals with baseline BMI of 30 or greater, compared with the reference group with stable LCD indices, participants in Q5 of HLCD had 1.63 (95% CI, 1.40 to 1.86) kg less weight gain (BMI <25: −0.39 [95% CI, −0.44 to −0.35] kg less weight gain), whereas the Q5 of ULCD was associated with 0.67 (95% CI, 0.44 to 0.90) kg more weight gain (BMI <25: 0.33 [95% CI, 0.28 to 0.37] kg more weight gain). Figure 2 shows the associations of changing LCDS with weight change by baseline BMI. The pattern of associations was independent of the choice of weight change measurements (ie, weight change in kg, percentage of weight change, or changes in BMI).

**Table 3.** Weight Changes Over 4-Year Periods According to 4-Year Change in LCD Scores, Stratified by Selected Characteristics^a^

Characteristic	Weight change, mean (95% CI), kg					*P* for interaction	Weight change per 1-SD, mean (95% CI), kg
	Q1	Q2	Q3	Q4	Q5		
**TLCD Score**							
Age, y							
<55	−0.23 (−0.29 to −0.17)	−0.04 (−0.09 to 0.02)	0 [Reference]	0.07 (0.01 to 0.12)	−0.07 (−0.13 to −0.01)	.20	0.05 (0.03 to 0.08)
≥55	−0.24 (−0.32 to −0.16)	−0.11 (−0.18 to −0.03)	0 [Reference]	0.06 (−0.01 to 0.14)	0.00 (−0.08 to 0.08)		0.09 (0.06 to 0.12)
Baseline BMI							
<25	−0.05 (−0.10 to −0.01)	−0.01 (−0.06 to 0.03)	0 [Reference]	0.06 (0.02 to 0.11)	0.02 (−0.02 to 0.07)	<.001	0.03 (0.01 to 0.04)
25 to <30	−0.42 (−0.52 to −0.31)	−0.14 (−0.24 to −0.04)	0 [Reference]	0.03 (−0.07 to 0.14)	−0.13 (−0.23 to −0.02)		0.10 (0.07 to 0.14)
≥30	−0.62 (−0.86 to −0.39)	−0.08 (−0.30 to 0.14)	0 [Reference]	0.24 (0.01 to 0.46)	−0.13 (−0.37 to 0.10)		0.17 (0.09 to 0.25)
AHEI							
<Median	−0.31 (−0.38 to −0.23)	−0.07 (−0.14 to −0.00)	0 [Reference]	0.05 (−0.02 to 0.12)	−0.18 (−0.26 to −0.11)	<.001	0.04 (0.02 to 0.07)
≥Median	−0.19 (−0.25 to −0.12)	−0.04 (−0.10 to 0.02)	0 [Reference]	0.07 (0.00 to 0.13)	0.02 (−0.05 to 0.08)		0.07 (0.05 to 0.10)
Physical activity							
<Median	−0.27 (−0.35 to −0.19)	−0.04 (−0.11 to 0.04)	0 [Reference]	0.06 (−0.01 to 0.13)	−0.13 (−0.21 to −0.05)	.047	0.04 (0.01 to 0.07)
≥Median	−0.26 (−0.32 to −0.20)	−0.09 (−0.15 to −0.03)	0 [Reference]	0.04 (−0.02 to 0.10)	−0.05 (−0.11 to 0.01)		0.08 (0.05 to 0.10)
**ALCD Score**							
Age, y							
<55	−0.25 (−0.31 to −0.19)	−0.03 (−0.09 to 0.02)	0 [Reference]	0.07 (0.01 to 0.13)	0.08 (0.02 to 0.14)	.35	0.12 (0.10 to 0.14)
≥55	−0.27 (−0.35 to −0.19)	−0.07 (−0.15 to 0.01)	0 [Reference]	0.11 (0.03 to 0.18)	0.08 (−0.00 to 0.16)		0.14 (0.11 to 0.17)
Baseline BMI							
<25	−0.08 (−0.13 to −0.03)	0.01 (−0.04 to 0.05)	0 [Reference]	0.11 (0.07 to 0.15)	0.17 (0.12 to 0.21)	<.001	0.09 (0.07 to 0.11)
25 to <30	−0.38 (−0.49 to −0.28)	−0.06 (−0.16 to 0.04)	0 [Reference]	0.12 (0.02 to 0.22)	0.03 (−0.08 to 0.13)		0.15 (0.12 to 0.19)
≥30	−0.88 (−1.11 to −0.64)	−0.28 (−0.52 to −0.05)	0 [Reference]	−0.09 (−0.32 to 0.14)	−0.14 (−0.38 to 0.09)		0.26 (0.18 to 0.34)
AHEI							
<Median	−0.34 (−0.41 to −0.27)	−0.08 (−0.15 to −0.01)	0 [Reference]	0.00 (−0.07 to 0.07)	−0.07 (−0.15 to 0.00)	<.001	0.10 (0.08 to 0.13)
≥Median	−0.19 (−0.25 to −0.12)	−0.00 (−0.06 to 0.06)	0 [Reference]	0.13 (0.07 to 0.19)	0.15 (0.09 to 0.22)		0.12 (0.10 to 0.14)
Physical activity							
<Median	−0.31 (−0.38 to −0.23)	−0.05 (−0.13 to 0.02)	0 [Reference]	0.00 (−0.07 to 0.08)	−0.06 (−0.14 to 0.02)	.003	0.09 (0.06 to 0.12)
≥Median	−0.25 (−0.31 to −0.18)	−0.05 (−0.11 to 0.01)	0 [Reference]	0.11 (0.05 to 0.17)	0.11 (0.05 to 0.17)		0.13 (0.11 to 0.15)
**VLCD Score**							
Age, y							
<55	−0.18 (−0.24 to −0.13)	−0.06 (−0.12 to −0.01)	0 [Reference]	−0.06 (−0.12 to −0.01)	−0.26 (−0.31 to −0.20)	.01	−0.04 (−0.06 to −0.02)
≥55	−0.22 (−0.30 to −0.14)	−0.09 (−0.17 to −0.02)	0 [Reference]	−0.03 (−0.10 to 0.05)	−0.12 (−0.20 to −0.04)		0.02 (−0.01 to 0.05)
Baseline BMI							
<25	−0.05 (−0.10 to −0.01)	−0.03 (−0.07 to 0.02)	0 [Reference]	−0.08 (−0.13 to −0.04)	−0.17 (−0.22 to −0.13)	<.001	−0.05 (−0.07 to −0.04)
25 to <30	−0.32 (−0.42 to −0.22)	−0.13 (−0.23 to −0.03)	0 [Reference]	−0.02 (−0.12 to 0.08)	−0.22 (−0.32 to −0.12)		0.03 (−0.01 to 0.07)
≥30	−0.55 (−0.77 to −0.32)	−0.18 (−0.40 to 0.03)	0 [Reference]	0.12 (−0.10 to 0.34)	−0.48 (−0.71 to −0.26)		0.04 (−0.05 to 0.12)
AHEI							
<Median	−0.26 (−0.34 to −0.19)	−0.09 (−0.15 to −0.02)	0 [Reference]	−0.07 (−0.14 to −0.00)	−0.36 (−0.43 to −0.29)	<.001	−0.06 (−0.09 to −0.04)
≥Median	−0.18 (−0.24 to −0.12)	−0.08 (−0.14 to −0.02)	0 [Reference]	−0.04 (−0.10 to 0.02)	−0.10 (−0.16 to −0.03)		0.03 (0.01 to 0.05)
Physical activity							
<Median	−0.25 (−0.33 to −0.18)	−0.12 (−0.19 to −0.05)	0 [Reference]	−0.06 (−0.13, 0.01)	−0.28 (−0.36, −0.21)	.33	−0.02 (−0.05 to 0.01)
≥Median	−0.19 (−0.25 to −0.13)	−0.07 (−0.12 to −0.01)	0 [Reference]	−0.04 (−0.10, 0.02)	−0.17 (−0.22, −0.11)		−0.00 (−0.02 to 0.02)
**HLCD Score**							
Age, y							
<55	0.38 (0.32 to 0.43)	0.18 (0.12 to 0.23)	0 [Reference]	−0.21 (−0.26 to −0.15)	−0.71 (−0.77 to −0.65)	<.001	−0.41 (−0.43 to −0.39)
≥55	0.13 (0.05 to 0.21)	0.08 (0.01 to 0.16)	0 [Reference]	−0.15 (−0.23 to −0.08)	−0.48 (−0.56 to −0.40)		−0.23 (−0.26 to −0.20)
Baseline BMI							
<25	0.19 (0.15 to 0.24)	0.07 (0.03 to 0.11)	0 [Reference]	−0.15 (−0.20 to −0.11)	−0.39 (−0.44 to −0.35)	<.001	−0.23 (−0.24 to −0.21)
25 to <30	0.40 (0.29 to 0.50)	0.26 (0.17 to 0.36)	0 [Reference]	−0.19 (−0.29 to −0.09)	−0.84 (−0.94 to −0.74)		−0.45 (−0.49 to −0.41)
≥30	0.75 (0.53 to 0.97)	0.40 (0.18 to 0.61)	0 [Reference]	−0.42 (−0.64 to −0.20)	−1.63 (−1.86 to −1.40)		−0.88 (−0.97 to −0.80)
AHEI							
<Median	0.24 (0.17 to 0.32)	0.15 (0.09 to 0.22)	0 [Reference]	−0.17 (−0.24 to −0.11)	−0.75 (−0.82 to −0.68)	<.001	−0.41 (−0.44 to −0.38)
≥Median	0.31 (0.25 to 0.37)	0.14 (0.08 to 0.20)	0 [Reference]	−0.21 (−0.27 to −0.15)	−0.54 (−0.61 to −0.48)		−0.30 (−0.32 to −0.28)
Physical activity							
<Median	0.38 (0.30 to 0.45)	0.17 (0.10 to 0.24)	0 [Reference]	−0.22 (−0.29 to −0.15)	−0.79 (−0.86 to −0.71)	<.001	−0.43 (−0.46 to −0.40)
≥Median	0.20 (0.14 to 0.26)	0.11 (0.05 to 0.17)	0 [Reference]	−0.17 (−0.23 to −0.11)	−0.53 (−0.59 to −0.47)		−0.28 (−0.30 to −0.26)
**ULCD Score**							
Age, y							
<55	−0.69 (−0.74 to −0.63)	−0.20 (−0.26 to −0.15)	0 [Reference]	0.19 (0.13 to 0.24)	0.42 (0.36 to 0.48)	<.001	0.41 (0.39 to 0.43)
≥55	−0.48 (−0.56 to −0.40)	−0.06 (−0.14 to 0.01)	0 [Reference]	0.28 (0.21 to 0.36)	0.33 (0.25 to 0.41)		0.31 (0.28 to 0.34)
Baseline BMI							
<25	−0.28 (−0.32 to −0.23)	−0.06 (−0.10 to −0.02)	0 [Reference]	0.21 (0.17 to 0.25)	0.33 (0.28 to 0.37)	<.001	0.23 (0.21 to 0.25)
25 to <30	−0.88 (−0.98 to −0.78)	−0.19 (−0.29 to −0.09)	0 [Reference]	0.24 (0.14 to 0.34)	0.49 (0.39 to 0.60)		0.50 (0.46 to 0.54)
≥30	−1.78 (−2.01 to −1.56)	−0.60 (−0.82 to −0.37)	0 [Reference]	0.31 (0.10 to 0.53)	0.67 (0.44 to 0.90)		0.92 (0.84 to 1.01)
AHEI							
<Median	−0.74 (−0.81 to −0.67)	−0.21 (−0.28 to −0.14)	0 [Reference]	0.13 (0.06 to 0.20)	0.20 (0.13 to 0.28)	<.001	0.38 (0.36 to 0.41)
≥Median	−0.52 (−0.59 to −0.45)	−0.10 (−0.16 to −0.04)	0 [Reference]	0.29 (0.23 to 0.35)	0.48 (0.42 to 0.54)		0.35 (0.33 to 0.38)
Physical activity							
<Median	−0.73 (−0.81 to −0.65)	−0.15 (−0.22 to −0.07)	0 [Reference]	0.21 (0.14 to 0.28)	0.36 (0.28 to 0.44)	.001	0.40 (0.38 to 0.43)
≥Median	−0.56 (−0.62 to −0.50)	−0.16 (−0.22 to −0.10)	0 [Reference]	0.21 (0.15 to 0.27)	0.35 (0.29 to 0.41)		0.34 (0.31 to 0.36)

Abbreviations: AHEI, Alternative Healthy Eating Index–2010; ALCD, animal-based low-carbohydrate diet; BMI, body mass index (calculated as weight in kilograms divided by height in meters squared); HLCD, healthy low-carbohydrate diet; Q1, quintile 1 (large decrease); Q2, quintile 2 (moderate decrease); Q3, quintile 3 (no change); Q4, quintile 4 (moderate increase); Q5, quintile 5 (large increase); TLCD, total low-carbohydrate diet; ULCD, unhealthy low-carbohydrate diet; VLCD, vegetable-based low-carbohydrate diet.

^a^ Multivariable adjusted models adjusted for age, race and ethnicity, family history of diabetes, baseline hypertension, baseline hypercholesterolemia, baseline total caloric intake, baseline body mass index, change in smoking status, baseline and change in physical activity, change in alcohol consumption, postmenopausal hormone use (women only), oral contraceptive use (NHSII only). Pooled results were further adjusted for cohort.

## Discussion

This cohort study investigated the associations between changes in various LCD scores and weight change in 3 large prospective cohorts of US men and women. We observed an inverted U-shape of the associations between changes in TLCDs with weight change over 4-year periods. However, when we further differentiated between the sources and quality of macronutrients when constructing the LCD indices, we found divergent associations with weight changes in that the LCDs that emphasize the intake of high-quality macronutrients from healthy plant-based foods were associated with less weight gain, whereas LCDs that emphasize animal-sourced proteins and fats or refined carbohydrates were associated with more weight gain. These associations were more apparent among younger, heavier, and less active individuals.

The association between LCDs and weight change has been extensively explored in randomized clinical trials (RCTs). The Dietary Intervention Randomized Controlled Trial^21^ demonstrated that a diet composed of 20% carbohydrates resulted in a significant weight reduction of 4.7 kg over a 24-month span compared with a Mediterranean diet (4.4 kg) and a low-fat diet (2.9 kg). The A to Z trial^22^ reported similar findings, with the Atkins group (<10% carbohydrates) losing more weight than the other diet groups with 40% or more carbohydrates. However, in the DIETFITS trial that compared healthy low-fat (48% carbohydrates) and healthy low-carbohydrate (30% carbohydrates) diets, there was no significant difference in weight change between groups.^23^ Similarly, a 2009 RCT by Sacks et al^24^ showed no significant differences in weight loss between diets with 35% and 65% carbohydrates. Observational studies also provide valuable insights. In the EPIC-PANACEA study (daily carbohydrate intake of 30% to 50% of energy), an iso-energetic increase of 5% of energy from protein at the expense of 5% of energy from carbohydrates was associated with 0.4 kg weight gain in men and 0.6 kg weight gain in women.^25^ Another study with data from 3 prospective cohorts (median daily carbohydrates intake, 44% to 49% of energy) did not find a significant association between carbohydrate intake and weight gain.^26^ The reasons behind these mixed findings from RCTs and observational studies are unknown; however, the percentage of energy obtained from carbohydrates in these diets, as well as the differences in diet quality, may be key factors.

Our study further distinguished between the quality of macronutrients in LCDs. We identified disparate trends between ALCDs and VLCDs, and likewise between ULCDs and HLCDs. Earlier studies have robustly demonstrated the role of food quality, not just the macronutrient quantity or composition, in influencing weight outcomes. In a previous study in the NHS, NHSII, and HPFS,^6^ we reported that increasing daily intakes of red and processed meats, potatoes, and sugar-sweetened beverages were associated with long-term weight gain, while increasing intakes of vegetables, whole grains, fruits, nuts, and yogurt were associated with less weight gain, emphasizing the significance of quality and not just quantity of food in weight outcomes. Consistently, the PREDIMED trial documented that a high-fat (41.2% fat, 40.4% carbohydrates), extra-virgin olive oil–supplemented Mediterranean diet, rich in fruits, vegetables, whole grains, and legumes, significantly reduced weight by 0.4 kg on average, compared to a low-fat diet (37.0% fat, 43.7% carbohydrates).^27^ Moreover, the PREDIMED-Plus trial^28^ combined the energy-restricted Mediterranean diet (37.2% carbohydrates, with a special emphasis of carbohydrate intakes from whole grains) with physical activity over a year and found that participants experienced a mean weight loss of 3.2 kg, compared with 0.7 kg in the control group (39.3% carbohydrates). Our findings highlight the importance of considering macronutrient quality in LCDs when assessing their associations with weight change, further endorsing the emphasis on healthier food groups as an effective approach for weight management. Indeed, it is well established that higher fiber content can promote satiety and reduce overall calorie intake.^29^ Additionally, consuming unsaturated oils from plant sources, such as nuts, seeds, and olive oil, has been associated with better weight outcomes and reduced cardiometabolic risk compared with saturated fats from animal sources.^30,31,32^ Conversely, saturated fats have been associated with increased inflammation, insulin resistance, and higher risk of weight gain and obesity.^33,34,35,36^ Furthermore, consuming refined carbohydrates and added sugars can lead to rapid fluctuations in blood glucose levels, which may contribute to increased hunger and calorie intake.^37,38,39^

Our stratified analysis also suggested differential associations of LCDs based on participants’ age, BMI, physical activity levels, and overall diet quality. A meta-analysis by Johnston et al^40^ summarized diet-based weight loss RCTs among individuals who were overweight or obese and reported that LCDs (10% to 40% of energy from carbohydrates) were associated with an estimated 7.25 kg weight loss at 12-month follow-up compared with referenced diets.^40^ Of note, participants in our cohorts followed a diet with higher carbohydrate amounts than most RCTs, constituting 40% to 60% of energy, for an extended period. We observed that individuals who were obese with high adherence to a healthy LCD experienced 1.63 kg less weight gain compared with no change in diet. The corresponding effect estimate among participants with BMI within reference range (<25) was only 0.39 kg, suggesting the potential importance of factors, such as baseline BMI, at the individual level in weight loss outcomes. In fact, previous studies have suggested that weight loss attempts among lean individuals (BMI <25) may lead to unfavorable weight changes and other metabolic outcomes.^41^ For example, lean dieters may have a higher risk of type 2 diabetes, fat or weight overshooting, and loss of fat-free mass compared with dieters who are overweight or obese.^42^ The biological mechanisms underpinning the interactions among participants’ diet, baseline BMI, and weight outcomes may be multifaceted. It is plausible that individuals who are obese respond differently to LCDs due to altered metabolic states (eg, insulin resistance), which could affect how dietary carbohydrates are processed and stored.^23^ These together might partially explain why people with different baseline body weights may respond differently to LCDs.

This study has several notable strengths. We created 5 versions of LCDs based on the macronutrient quality and amount, which provides a broader scope of examining the associations of LCDs with weight change. Second, the cohorts’ large sample sizes and long-term follow-up allowed us to explore the association between LCDs and weight change with relatively large statistical power.

### Limitations

Our study has some limitations. Given the self-reported nature of the data, measurement errors are inevitable, which could have led to misclassifications in diet and weight changes. Nevertheless, the validity of our dietary and weight data has been previously demonstrated. Furthermore, as in all observational studies, residual or unmeasured confounding could exist. Moreover, our study did not measure body composition, such as abdominal adiposity, so we were unable to ascertain how LCDs were associated with lean body mass and adiposity. Additionally, our study population included mainly White health professionals, which could limit generalizability.

## Conclusions

The findings of this cohort study underscore the importance of diet quality within LCD patterns for weight management. A high-quality LCD, rich in plant-based proteins and healthy fats, was associated with slower weight gain, while a lower-quality LCD was associated with the opposite result. Overall, the study findings argue against the sole focus of macronutrient quantity for weight management and suggest the crucial role of nutrient quality in maintaining a healthy body weight. Future studies should validate these findings in more diverse populations and elucidate the mechanisms underlying these associations.

## Notes

Supplement 1.
eMethods.
**eTable 1.** Age-Standardized Characteristics of Participants at Baseline, Stratified by Quintiles of Total Low-Carbohydrate Diet Score (TLCDS) at Baseline **eTable 2.** Age-Standardized Characteristics of Participants at Baseline in Combined Dataset, Stratified by Quintiles of Each Low-Carbohydrate Diet Score at Baseline **eTable 3.** Spearman’s Rank Correlation Coefficients Between Alternative Healthy Eating Index-2010 With Each Low-Carbohydrate Diet Scores at Study Baseline **eTable 4.** Averages of Major Food Group Consumptions Comparing Extreme Quintiles of AHEI and Each Low-Carbohydrate Diet Score **eTable 5.** Comparison Between Independent Correlation Matrix vs Unstructured Correlation Matrix

Supplement 2.
Data Sharing Statement
