# Error in Figure 2

**DOI:** 10.1001/jamanetworkopen.2024.0829

**Published:** 2024-02-08

In the Original Investigation titled “Low-Carbohydrate Diet Macronutrient Quality and Weight Change,”^1^ published December 27, 2023, Figure 2 should have specified that changes were measured per 1-SD change in diet score. This article has been corrected.^1^
