# Supplementary material for: Low-Carbohydrate Diet Macronutrient Quality and Weight Change
Source: JAMA Netw Open. 2023 Dec 27;6(12):e2349552. doi: 10.1001/jamanetworkopen.2023.49552 (PMC10753393; doi:10.1001/jamanetworkopen.2023.49552)
Supplement: Supplement 1. — eMethods. eTable 1. Age-Standardized Characteristics of Participants at Baseline, Stratified by Quintiles of Total Low-Carbohydrate Diet Score (TLCDS) at Baseline eTable 2. Age-Standardized Characteristics of Participants at Baseline in Combined Dataset, Stratified by Quintiles of Each Low-Carbohydrate Diet Score at Baseline eTable 3. Spearman’s Rank Correlation Coefficients Between Alternative Healthy Eating Index-2010 With Each Low-Carbohydrate Diet Scores at Study Baseline eTable 4. Averages of Major Food Group Consumptions Comparing Extreme Quintiles of AHEI and Each Low-Carbohydrate Diet Score eTable 5. Comparison Between Independent Correlation Matrix vs Unstructured Correlation Matrix [file jamanetwopen-e2349552-s001.pdf]

Supplemental Online Content

Liu B, Hu Y, Rai SK, Wang M, Hu FB, Sun Q. Low-carbohydrate diet macronutrient quality and weight change. *JAMA Netw Open*. 2023;6(12):e2349552. doi:10.1001/jamanetworkopen.2023.49552

eMethods.
eTable 1. Age-Standardized Characteristics of Participants at Baseline, Stratified by Quintiles of Total Low-Carbohydrate Diet Score (TLCDS) at Baseline
eTable 2. Age-Standardized Characteristics of Participants at Baseline in Combined Dataset, Stratified by Quintiles of each Low-Carbohydrate Diet Score at Baseline
eTable 3. Spearman's Rank Correlation Coefficients Between Alternative Healthy Eating Index-2010 With Each Low-Carbohydrate Diet Scores at Study Baseline
eTable 4. Averages of Major Food Group Consumptions Comparing Extreme Quintiles of AHEI and Each Low-Carbohydrate Diet Score
eTable 5. Comparison Between Independent Correlation Matrix vs Unstructured Correlation Matrix

This supplemental material has been provided by the authors to give readers additional information about their work.

© 2023 Liu B et al. *JAMA Network Open.*

eMethods

Baseline Exclusion criteria for diseases in detail

At baseline (1986 for NHS and HPFS, and 1991 for NHSII), we excluded participants with self-reported diabetes, cardiovascular disease (myocardial infarction, coronary artery surgery, stroke, pulmonary embolism), cancer (except nonmelanoma skin cancer), respiratory diseases (emphysema, active tuberculosis, chronic bronchitis), neurodegenerative disorders (amyotrophic lateral sclerosis, Parkinson's disease, multiple sclerosis, Alzheimer's disease), gastric conditions (ulcerative colitis, gastric or duodenal ulcers, gastric surgery/intestinal bypass), chronic kidney disease, or systemic lupus erythematosus as these conditions may lead to substantial weight changes.

Computation of Low carbohydrate diet (LCD) scores

Consumption levels of carbohydrates, fat, and protein were ranked into 11 categories based on their percentage contributions to total energy intake. Fat and protein intake were ranked in ascending order, scoring from 0 (lowest intake) to 10 (highest intake), while carbohydrate intake was ranked in the descending order, scoring from 10 (lowest intake) to 0 (highest intake). We summed the ranks of these three macronutrients to derive the total LCD score (TLCDS). An animal-based LCD score (ALCDS) and a vegetable-based LCD score (VLCDS) were created using the same algorithm, except that animal sources of fat/protein and plant sources of fat/protein were ranked, respectively. A healthy LCD (HLCD) and an unhealthy LCD (ULCD) were derived to further consider the quality of carbohydrates. The HLCDS was calculated by summing the ranks of vegetable protein and fat and a reversed rank of less healthful carbohydrates, including those from potato, added sugar, and refined grains. The ULCDS was calculated by summing the ranks of animal protein and fat, and a reversed rank of healthful carbohydrates, including those from non-starchy vegetables (excluding potatoes and French fries), fruits (excluding added sugar from fruit juice), legumes, and whole grains. By reversing the ranking of refined versus whole grain carbohydrates, the carbohydrate composition of the HLCDS and ULCDS reflects the special emphasis of whole grains and refined carbohydrates, respectively.

© 2023 Liu B et al. *JAMA Network Open.*

Assessment of Covariates

Information on participants' demographic and lifestyle factors, as well as the occurrence of new medical diagnoses, was collected biennially through a validated questionnaire in these three cohorts since baseline. This included age, self-reported races and ethnicities (assessed as basic demographic variables; African American, Asian, White, Hispanic and Other), smoking status, physical activity, family history of diabetes, hypertension and hypercholesterolemia at baseline, postmenopausal hormone use (women only), and oral contraceptive use (NHSII only). Alcohol consumption and total caloric intake were measured from FFQ every 4 years. Physical activity was measured using a validated questionnaire,[1–3] which derived metabolic equivalent tasks in hours per week based on time spent on 10 recreational activities. Height was measured in the first questionnaire of these three cohorts, and time-varying body mass index (BMI) was calculated as biennially updated weight in kilograms divided by the square of height in meters. The Alternative Healthy Eating Index-2010 (AHEI) was calculated based on participants' FFQ responses to reflect overall diet quality. To maximize the power of the analysis, missing values of physical activity, alcohol intake, total calories intake, and AHEI during the follow-up were replaced by the values in the previous questionnaire cycle.

Confounding adjustment in Statistical models

All multivariable models adjusted for age, races and ethnicities (African American, Asian, White, Hispanic and Other, or missing), family history of diabetes (yes/no), baseline hypertension (yes/no), baseline hypercholesterolemia (yes/no), baseline total caloric intake (continuous, kcal/day), baseline BMI (<25.0, 25.0-29.9, or ≥30.0 kg/m^2), change in smoking status (stayed never smoker, stayed former smoker, stayed current smoker, change from former to current smoker, change from never to current smoker, and change from current to former smoker), baseline and change in physical activity (continuous, METs-hour/week), change in alcohol consumption (continuous, g/day), postmenopausal hormone use (women only; premenopausal, never, former, current, or missing), and oral contraceptive use (NHSII only; yes, no).

© 2023 Liu B et al. *JAMA Network Open.*

References

1. Ainsworth BE, Haskell WL, Leon AS, et al. Compendium of Physical Activities: classification of energy costs of human physical activities. *Medicine & Science in Sports & Exercise*. 1993;25(1):71.
2. Chasan-Taber S, Rimm EB, Stampfer MJ, et al. Reproducibility and validity of a self-administered physical activity questionnaire for male health professionals. *Epidemiology*. 1996;7(1):81-86. doi:10.1097/00001648-199601000-00014
3. Wolf AM, Hunter DJ, Colditz GA, et al. Reproducibility and validity of a self-administered physical activity questionnaire. *Int J Epidemiol*. 1994;23(5):991-999. doi:10.1093/ije/23.5.991

© 2023 Liu B et al. *JAMA Network Open.*

eTable 1. Age-standardized characteristics of participants at baseline, stratified by quintiles of total low-carbohydrate diet score (TLCDS) at baseline.

NHS (N=47458)	**Baseline Total Low-Carbohydrate Diet Score**				
	**Q1**	**Q2**	**Q3**	**Q4**	**Q5**
No. of participants	9601	8356	11436	8348	9717
Total Low Carbohydrate Diet Score	5 (3,7)	11 (10,12)	15 (14,16)	19 (18,20)	25 (23,27)
Animal-based Low Carbohydrate Diet Score	5 (2,7)	10 (8,13)	15 (12,18)	20 (17,22)	25 (23,28)
Plant-based Low Carbohydrate Diet Score	11 (8,15)	14 (10,17)	16 (12,19)	17 (13,21)	18 (14,21)
High-quality Low Carbohydrate Diet Score	13 (9,17)	15 (11,18)	15 (12,19)	16 (12,19)	16 (13,20)
Low-quality Low Carbohydrate Diet Score	7 (4,11)	11 (9,15)	15 (12,18)	18 (15,21)	23 (20,26)
Age (year)	52.9 (7.2)	52.5 (7.1)	52.2 (7.1)	51.8 (7.1)	51.5 (7.0)
Races and ethnicities (%)					
- White	79.1	81.2	81.4	81.4	80.8
- Asian	1.2	0.8	0.6	0.4	0.4
- Hispanic	0.6	0.5	0.5	0.4	0.3
- African American	1.4	0.9	0.7	0.6	0.6
- Missing	17.7	16.7	16.8	17.3	17.8
Current Smoking (%)	16.7	16.5	18.7	20.6	23.1
Alcohol intake (g/day)	5 (9.3)	6.9 (12.8)	7.2 (11.6)	7.2 (10.7)	5.5 (8.3)
Physical activity (METs-h/week)	16 (21.8)	15.4 (21.3)	14.5 (20)	13.4 (20)	12.5 (19.2)
Total energy intake (kcal/day)	1812 (530)	1788 (513)	1784 (512)	1770 (506)	1684 (510)
Weight (kg)	65.5 (12.3)	66.4 (12.3)	67.4 (12.9)	68.3 (13.3)	69.5 (14.0)
BMI (kg/m^2)	24.3 (4.2)	24.6 (4.3)	25 (4.5)	25.3 (4.6)	25.8 (4.9)
Alternative Healthy Eating Index-2010	52.9 (11.7)	53.2 (11.7)	52.4 (11.3)	51.9 (11.4)	51.9 (10.9)
Carbohydrates intake (% of energy)	57.2 (55,60.3)	52.4 (50.9,53.8)	48.8 (47.0,50.1)	45.2 (43.1,46.7)	40.4 (37.1,42.8)
- High-quality carbohydrates intake [#]	17.0 (12.3,22.5)	15.8 (11.8,20.1)	14.1 (10.8,17.9)	12.8 (9.7,16.2)	11.5 (8.9,14.6)
- Low-quality carbohydrates intake [#]	26.4 (22.2,31.0)	23.7 (19.8,27.6)	22.5 (19.0,26.2)	21.4 (17.9,24.6)	18.9 (15.6,22.2)

© 2023 Liu B et al. *JAMA Network Open.*

Protein intake (% of energy)	15.9 (14.4,17.3)	17.5 (15.9,19.3)	18.3 (16.7,20.1)	19.1 (17.5,20.9)	21.1 (19.5,22.9)
- Animal-based protein intake	10.5 (9.1,11.9)	12.3 (10.8,14.0)	13.2 (11.6,15.1)	14.2 (12.7,16.0)	16.4 (14.8,18.4)
- Plant-based protein intake	5.3 (4.6,6.0)	5.2 (4.6,5.9)	5.0 (4.5,5.6)	4.8 (4.3,5.4)	4.5 (4.0,5.1)
Fat intake (% of energy)	27.5 (24.7,29.9)	30.6 (27.7,33.0)	32.8 (30.2,35.3)	35.1 (32.5,37.8)	38.1 (35.6,40.8)
- Animal-based fat intake	13.8 (11.6,16.0)	16.1 (14.0,18.2)	17.9 (15.9,20.1)	20.0 (17.8,22.3)	23.3 (20.7,26.2)
- Plant-based fat intake	13.1 (10.6,15.7)	13.7 (11.1,16.6)	14.3 (11.6,17.3)	14.7 (11.8,17.9)	14.4 (11.6,17.5)
Baseline hypertension (%)	21.9	21.6	22.1	21.6	23.2
Baseline hypercholesterolemia (%)	12.6	11.4	10.6	10.3	10.1
Family history of diabetes (%)	24.7	25.1	25.7	25.6	27.0
Any use of postmenopausal hormone (%)	26.0	27.1	26.6	27.2	26.6
NHSII (N=55862) *	**Baseline Total Low-Carbohydrate Diet Score**				
	**Q1**	**Q2**	**Q3**	**Q4**	**Q5**
No. of participants	12024	11297	10078	12121	10342
Total Low Carbohydrate Diet Score	5 (2,7)	11 (10,12)	16 (15,17)	20 (19,21)	25 (24,27)
Animal-based Low Carbohydrate Diet Score	4 (2,7)	11 (8,13)	16 (13,18)	20 (18,22)	26 (24,28)
Plant-based Low Carbohydrate Diet Score	11 (7,15)	14 (10,17)	16 (12,19)	18 (14,21)	18 (15,22)
High-quality Low Carbohydrate Diet Score	13 (8,17)	15 (11,19)	16 (12,19)	17 (13,20)	17 (14,20)
Low-quality Low Carbohydrate Diet Score	7 (4,11)	12 (9,15)	15 (12,18)	19 (16,21)	23 (20,26)
Age (year)	36.7 (4.6)	36.9 (4.4)	36.9 (4.4)	37 (4.4)	37 (4.4)
Races and ethnicities (%)					
- White	91.4	93.6	93.8	94.5	93.4
- Asian	2.3	1.3	1.1	0.8	0.9
- Hispanic	1.4	1.1	1.3	0.8	1.2
- African American	1.8	1.0	1.0	1.0	1.2
- Missing	3.2	3.0	2.8	2.9	3.3
Current Smoking (%)	10.3	9.5	10.9	13.1	15.5
Alcohol intake (g/day)	2.8 (5.5)	3.5 (7.2)	3.5 (6.8)	3.6 (6.2)	2.8 (5.1)

© 2023 Liu B et al. *JAMA Network Open.*

Physical activity (METs-h/week)	24.6 (33.3)	22 (27)	20.2 (25.4)	18.7 (23.8)	16.9 (22.7)
Total energy intake (kcal/day)	1849 (556)	1816 (525)	1776 (509)	1746 (524)	1640 (507)
Weight (kg)	63.4 (13.1)	65.2 (13.9)	66.5 (14.1)	68.2 (15.6)	70.5 (16.9)
BMI (kg/m^2)	23.3 (4.5)	23.9 (4.8)	24.4 (4.9)	25 (5.4)	25.9 (5.9)
Alternative Healthy Eating Index-2010	49.3 (12)	48.8 (11.2)	47.9 (10.7)	47.4 (10.2)	47.2 (9.6)
Carbohydrates intake (% of energy)	58.6 (56.3,62)	52.9 (51.6,54.2)	49.5 (48.4,50.4)	45.9 (44.3,47.1)	40.9 (38,42.9)
- High-quality carbohydrates intake [#]	14.6 (10.2,19.9)	13.7 (10.3,17.5)	12.5 (9.5,15.7)	11.2 (8.6,14.2)	9.9 (7.6,12.5)
- Low-quality carbohydrates intake [#]	31.8 (26.7,37.9)	27.8 (23.8,31.9)	26.3 (22.7,30.1)	24.9 (21.6,28.1)	22.2 (19,25.2)
Protein intake (% of energy)	16.3 (14.6,17.8)	18.6 (16.8,20.3)	19.3 (17.5,21.3)	20 (18.3,22)	22.1 (20.5,24)
- Animal-based protein intake	10.9 (9.2,12.4)	13.3 (11.6,15)	14.3 (12.5,16.2)	15.1 (13.5,17.1)	17.6 (15.9,19.6)
- Plant-based protein intake	5.3 (4.5,6.3)	5.2 (4.6,5.8)	5 (4.5,5.6)	4.8 (4.3,5.3)	4.5 (3.9,5)
Fat intake (% of energy)	26.3 (23.4,28.6)	29.7 (26.9,32.1)	31.8 (29.3,34.4)	34.1 (31.6,36.9)	37.1 (34.8,39.8)
- Animal-based fat intake	13 (10.8,15)	15.6 (13.8,17.4)	17.3 (15.6,19)	19.1 (17.2,21.1)	22.2 (19.9,24.7)
- Plant-based fat intake	12.7 (10.6,15.1)	13.5 (11,16.1)	14.1 (11.6,16.9)	14.8 (12.2,17.7)	14.7 (12.2,17.4)
Baseline hypertension (%)	5.4	5.2	5.9	6.4	7.7
Baseline hypercholesterolemia (%)	14.1	13.8	14.4	13.9	15.6
Family history of diabetes (%)	15.3	14.9	15.7	16.9	18.7
Any use of postmenopausal hormone (%)	3.4	3.0	3.1	2.9	3.1
Use of oral contraceptives (%)	17.8	16.2	15.7	15.0	13.9
HPFS (N=20012)	**Baseline Total Low-Carbohydrate Diet Score**				
	**Q1**	**Q2**	**Q3**	**Q4**	**Q5**
No. of participants	3934	3667	3932	4484	3995
Total Low Carbohydrate Diet Score	5 (3,7)	11 (10,12)	14 (14,15)	19 (18,20)	24 (23,27)
Animal-based Low Carbohydrate Diet Score	5 (2,7)	10 (8,13)	15 (12,17)	20 (17,22)	25 (23,28)
Plant-based Low Carbohydrate Diet Score	12 (9,15)	14 (10,17)	15 (12,19)	17 (13,20)	17 (14,21)
High-quality Low Carbohydrate Diet Score	13 (10,17)	14 (11,18)	15 (11,18)	15 (12,19)	15 (12,20)
Low-quality Low Carbohydrate Diet Score	7 (4,11)	12 (9,15)	15 (12,18)	19 (16,21)	23 (20,26)

© 2023 Liu B et al. *JAMA Network Open.*

Age (year)	50 (7.9)	50.2 (7.8)	50.8 (7.8)	51 (7.8)	51 (7.7)
Races and ethnicities (%)					
- White	89.8	91.2	92.0	92.2	92.0
- Asian	2.2	1.3	1.3	1.1	1.2
- Other	2.7	2.2	1.8	1.8	1.8
- African American	1.2	0.9	0.6	0.5	0.5
- Missing	4.2	4.3	4.3	4.4	4.5
Current Smoking (%)	5.2	6.7	7.6	9.0	10.2
Alcohol intake (g/day)	9.8 (14.1)	12.5 (17.7)	13.2 (16.6)	12.3 (14.9)	9.6 (11.3)
Physical activity (METs-h/week)	25.7 (29.5)	23.1 (26.1)	20.8 (23.4)	19.6 (22.9)	17.2 (21.1)
Total energy intake (kcal/day)	2035.6 (613.6)	2016.1 (595.6)	2029.5 (602.9)	2031.3 (607.9)	1934.8 (605.3)
Weight (kg)	78.6 (10.6)	79.5 (10.5)	81.1 (11)	82.3 (11.4)	83.9 (12.3)
BMI (kg/m^2)	24.6 (2.8)	24.8 (2.8)	25.3 (2.9)	25.6 (3.1)	26.2 (3.5)
Alternative Healthy Eating Index-2010	54 (11.9)	53 (11.8)	51.6 (11.6)	51.1 (11.1)	51.2 (10.8)
Carbohydrates intake (% of energy)	56.8 (54.1,60.5)	51.2 (48.9,53.2)	47.7 (45.3,49.4)	43.8 (41.3,45.7)	38.3 (34.9,41.1)
- High-quality carbohydrates intake [#]	17.1 (12.1,23.3)	14.5 (10.4,19.4)	13 (9.6,16.9)	11.5 (8.6,15.1)	10.3 (7.6,13.2)
- Low-quality carbohydrates intake [#]	26.1 (21.4,31.3)	24.2 (19.9,28.4)	22.9 (18.8,26.9)	21.5 (17.9,25.1)	18.9 (15.6,22.1)
Protein intake (% of energy)	15.7 (14.1,17.2)	17.2 (15.6,19.1)	17.9 (16.4,19.8)	18.7 (17.3,20.6)	20.9 (19.4,22.8)
- Animal-based protein intake	10.3 (8.8,11.7)	12.1 (10.6,13.8)	13.1 (11.5,14.7)	14.1 (12.6,15.9)	16.5 (14.9,18.4)
- Plant-based protein intake	5.3 (4.6,6.2)	5.1 (4.4,5.8)	4.9 (4.3,5.5)	4.7 (4.1,5.3)	4.3 (3.8,5)
Fat intake (% of energy)	26.3 (23,29)	29.8 (26.5,32.4)	32.1 (29.3,34.6)	34.7 (32.1,37.4)	38.2 (35.7,41)
- Animal-based fat intake	13.4 (10.7,15.7)	16.1 (13.9,18.3)	18.1 (15.9,20.4)	20.6 (18.2,22.9)	24.1 (21.3,27.2)
- Plant-based fat intake	12.3 (9.9,14.8)	12.9 (10.2,15.7)	13.3 (10.8,16.1)	13.7 (11.1,16.7)	13.5 (10.9,16.7)
Baseline hypertension (%)	16.1	16.1	18.1	16.6	17.3
Baseline hypercholesterolemia (%)	11.7	10.8	10.3	8.7	8.6
Family history of diabetes (%)	19.4	19.3	19.4	21.0	22.5

© 2023 Liu B et al. *JAMA Network Open.*

*A total of 61949 participants in NHSII were included for data analysis, but a significant number of them were pregnant at baseline and were included in later cycles.
[#] High-quality carbohydrates include fruit carbohydrate excluding juice sugar, vegetable carbohydrate excluding potato, and sum of carbohydrate from whole grains. Low-quality carbohydrates include sum of carbohydrate from potato, added sugar in foods, and refined grains.
Note: All continuous values are Mean (SD), except for Low carbohydrate diet scores and macronutrients intake being shown in Median (Q25, Q75), dichotomous variables are shown in percentage. Baselines were 1986 for NHS, 1991 for NHSII, and 1986 for HPFS.
Abbreviations: TLCDS: total low-carbohydrate diet score; Q1-Q5: Quintile 1-5; NHS: Nurses' Health Study; NHSII: Nurses' Health Study II; HPFS: Health Professionals Follow-up Study; BMI: body mass index.

© 2023 Liu B et al. *JAMA Network Open.*

eTable 2. Age-standardized characteristics of participants at baseline in combined dataset, stratified by quintiles of each low-carbohydrate diet score at baseline (N=123332).

	TLCDS		ALCDS		VLCDS		HLCDS		ULCDS	
	**Q1**	**Q5**	**Q1**	**Q5**	**Q1**	**Q5**	**Q1**	**Q5**	**Q1**	**Q5**
No. of participants	25559	26258	24988	25028	25194	21416	24877	26280	22244	22974
Total Low Carbohydrate Diet Score	5 (3,7)	25 (23,27)	5 (3,8)	24 (22,27)	10 (5,14)	20 (16,23)	11 (6,17)	17 (12,22)	6 (3,9)	24 (21,27)
Animal-based Low Carbohydrate Diet Score	4 (2,7)	25 (23,28)	4 (2,6)	26 (24,28)	13 (7,19)	16 (11,21)	15 (8,21)	14 (8,20)	5 (2,7)	25 (23,28)
Plant-based Low Carbohydrate Diet Score	11 (8,15)	18 (14,21)	14 (10,18)	16 (12,19)	8 (6,10)	23 (21,24)	9 (6,11)	21 (19,24)	15 (11,18)	15 (12,19)
High-quality Low Carbohydrate Diet Score	13 (9,17)	16 (13,20)	15 (11,19)	14 (11,18)	9 (6,11)	21 (19,24)	8 (6,9)	22 (21,24)	18 (14,21)	13 (10,16)
Low-quality Low Carbohydrate Diet Score	7 (4,11)	23 (20,26)	6 (4,10)	23 (21,26)	14 (10,19)	16 (11,20)	17 (12,22)	12 (7,17)	5 (3,7)	24 (23,26)
Age (year)	44.8 (9.9)	44.5 (9.4)	44.9 (9.9)	44.6 (9.5)	44.9 (10)	44.8 (9.4)	44.2 (9.5)	45.6 (9.8)	45.9 (10.1)	44.2 (9.2)
Races and ethnicities (%)										
- White	86.3	88.5	86.8	88.1	85.9	89.5	85.6	89.7	87.5	88.0
- Asian	1.9	0.8	1.9	0.8	1.6	0.9	1.8	0.8	1.8	0.8
- Hispanic or Other	1.3	0.9	1.2	1.0	1.2	0.8	1.0	1.1	1.4	0.9
- African American	1.5	0.9	1.3	1.0	1.8	0.4	1.6	0.7	1.3	0.9
- Missing	9.0	9.0	8.7	9.1	9.5	8.4	9.9	7.7	8.0	9.3
Current Smoking (%)	11.9	17.5	10.9	17.9	14.6	15.4	18.4	11.6	8.6	20.1
Alcohol intake (g/day)	4.8 (9.2)	5.0 (8.1)	4.2 (7.7)	5.9 (9.8)	5.1 (10.6)	5.7 (9.2)	6.0 (11.9)	5.4 (8.8)	5.7 (10.1)	5.2 (9.1)
Physical activity (METs-h/week)	21.4 (29.1)	15.3 (21.2)	21.5 (28.9)	15.5 (21.8)	19.2 (26.5)	17.1 (23.4)	16.1 (23.8)	20.6 (26.8)	25.1 (31.2)	13.7 (20.0)
Total energy intake (kcal/day)	1864 (561)	1709 (534)	1863 (566)	1704 (529)	1842 (550)	1774 (557)	1890 (566)	1738 (536)	1796 (541)	1757 (541)
Weight (kg)	66.6 (13.5)	72.1 (16.0)	66.6 (13.4)	72.1 (15.9)	68.5 (14.5)	70.1 (15.2)	69.3 (15.3)	69.0 (14.2)	66.1 (12.7)	72.3 (16.2)
BMI (kg/m^2)	23.9 (4.2)	25.9 (5.2)	23.9 (4.2)	25.9 (5.2)	24.6 (4.6)	25.1 (4.9)	24.8 (4.9)	24.7 (4.5)	23.7 (3.9)	25.8 (5.3)
Alternative Healthy Eating Index-2010	51.5 (12.0)	49.6 (10.6)	53.4 (12.2)	48.0 (10.1)	46.7 (10.9)	54.9 (11.2)	42.4 (9.2)	59.2 (10.4)	58.9 (10.5)	44.0 (9.2)
Carbohydrates intake (% of energy)	57.8 (55.5,61.2)	40.6 (37.4,43)	57.5 (54.5,61.2)	40.6 (37.2,43.3)	53.7 (49.3,58.3)	44.5 (41,47.8)	51.2 (46.0,56.3)	47.3 (42.7,51.8)	57.0 (53.4,60.9)	41.0 (37.3,44.2)
- High-quality carbohydrates intake [#]	15.9 (11.3,21.6)	10.6 (8.1,13.5)	16.2 (11.6,21.8)	10.6 (8,13.5)	13.1 (9.2,17.9)	12.2 (9.3,15.5)	9.9 (7.3,13.2)	16 (12.4,20.3)	19.8 (16.4,24.2)	8.6 (6.8,10.6)

© 2023 Liu B et al. *JAMA Network Open.*

- Low-quality carbohydrates intake [#]	28.6 (23.7,34.3)	20.6 (17.1,23.9)	28.6 (23.8,33.9)	20.3 (16.8,23.6)	25.5 (20.3,31.7)	23.6 (19.7,27.3)	28.8 (24.5,33.9)	20.7 (17.4,24.0)	24.9 (20.6,29.4)	22.5 (18.7,26.2)
Protein intake (% of energy)	16.1 (14.5,17.6)	21.4 (19.8,23.3)	15.8 (14.3,17.4)	21.8 (20.1,23.7)	18.3 (16.0,20.6)	18.6 (16.8,20.5)	17.6 (15.6,19.8)	19.1 (17.1,21.2)	16.8 (15.2,18.5)	20.8 (19.1,22.7)
- Animal-based protein intake	10.7 (9.1,12.1)	16.8 (15.2,18.9)	10.2 (8.7,11.5)	17.3 (15.8,19.3)	13.9 (11.6,16.3)	13.0 (11.0,15.0)	13.4 (11.3,15.7)	13.2 (11.1,15.5)	10.9 (9.2,12.4)	16.5 (14.8,18.5)
- Plant-based protein intake	5.3 (4.5,6.2)	4.5 (4,5.1.0)	5.7 (4.9,6.5)	4.3 (3.8,4.8)	4.3 (3.7,4.8)	5.6 (5.1,6.1)	4.1 (3.6,4.6)	5.8 (5.3,6.4)	6.0 (5.3,6.7)	4.2 (3.7,4.6)
Fat intake (% of energy)	26.8 (23.8,29.2)	37.5 (35,40.3)	27.4 (24.1,30.6)	36.6 (33.7,39.7)	28.2 (24.8,31.3)	36.6 (33.8,39.5)	30.4 (26.8,34.0)	33.6 (30.1,37.3)	26.8 (23.6,29.8)	37.3 (34.6,40.2)
- Animal-based fat intake	13.4 (11.1,15.5)	22.7 (20.1,25.6)	12.7 (10.7,14.4)	23.4 (21.3,26.0)	17.5 (14.6,20.8)	17.2 (14.6,19.9)	19 (15.7,22.5)	15.9 (13.2,18.7)	12.5 (10.5,14.3)	23.6 (21.4,26.2)
- Plant-based fat intake	12.8 (10.5,15.3)	14.4 (11.8,17.4)	14.6 (11.8,17.5)	12.6 (10.3,15.2)	10.0 (8.3,11.6)	18.9 (17.0,21.3)	10.9 (9.0,12.9)	17.3 (15.0,19.9)	14.0 (11.3,16.9)	13.2 (10.7,15.9)
Baseline hypertension (%)	13.6	15.1	13.1	15.4	14.5	13.3	14.6	13.3	12.9	14.8
Baseline hypercholesterolemia (%)	13.2	12.2	13.6	11.6	11.8	13.3	11.3	13.2	13.9	11.2
Family history of diabetes (%)	19.6	22.4	19.5	22.4	20.4	20.7	20.3	20.3	19.3	22.0

[#] High-quality carbohydrates include fruit carbohydrate excluding juice sugar, vegetable carbohydrate excluding potato, and sum of carbohydrate from whole grains. Low-quality carbohydrates include sum of carbohydrate from potato, added sugar in foods, and refined grains.
Note: All continuous values are Mean (SD), except for Low carbohydrate diet scores and macronutrients intake being shown in Median (Q25, Q75), dichotomous variables are shown in percentage. Baselines were 1986 for NHS, 1991 for NHSII, and 1986 for HPFS.
Abbreviations: TLCDS: total low-carbohydrate diet score; ALCDS: animal low-carbohydrate diet score; VLCDS: vegetable low-carbohydrate diet score; HLCDS: healthy low-carbohydrate diet score; ULCDS: unhealthy low-carbohydrate diet score; Q1, Q5: Quintile 1, Quintile 5 of the specified Low carbohydrate diet score; BMI: body mass index.

© 2023 Liu B et al. *JAMA Network Open.*

eTable 3. Spearman's Rank Correlation coefficients between Alternative Healthy Eating Index-2010 with each low-carbohydrate diet scores at study baseline (N=123332).

	**Alternative Healthy Eating Index-2010**
Total low-carbohydrate diet score	-0.06
Animal low-carbohydrate diet score	-0.16
Vegetable low-carbohydrate diet score	0.23
Healthy low-carbohydrate diet score	0.52
Unhealthy low-carbohydrate diet score	-0.45

Note: Baselines were 1986 for NHS, 1991 for NHSII, and 1986 for HPFS. All correlation coefficients have p<0.0001.

© 2023 Liu B et al. *JAMA Network Open.*

eTable 4. Averages of major food group consumptions comparing extreme quintiles of AHEI and each Low-carbohydrate diet score.

	AHEI		TLCDS		ALCDS		VLCDS		HLCDS		ULCDS	
**Unit: Servings/day**	**Q1**	**Q5**	**Q1**	**Q5**	**Q1**	**Q5**	**Q1**	**Q5**	**Q1**	**Q5**	**Q1**	**Q5**
Median score	38.5	69.6	5	25	4	26	8	22	8	23	5	24
Whole grains	0.9	1.6	1.6	0.9	1.7	0.8	1.1	1.3	0.9	1.7	1.9	0.7
*Starchy vegetables**	0.9	0.9	0.9	0.8	1.0	0.8	0.8	1.0	0.8	1.0	1.0	0.8
Non-starchy Vegetables	2.0	4.1	3.1	2.9	3.3	2.7	2.6	3.3	2.2	3.8	3.8	2.3
Dairy	2.3	2.1	2.0	2.3	1.9	2.5	2.7	1.8	2.5	2.0	1.8	2.6
Poultry	0.5	0.6	0.4	0.7	0.4	0.7	0.5	0.5	0.5	0.5	0.4	0.6
Seafood	0.2	0.4	0.2	0.3	0.2	0.3	0.3	0.3	0.2	0.3	0.3	0.3
Red and processed meat	1.3	0.5	0.6	1.3	0.5	1.4	0.9	0.8	1.1	0.7	0.4	1.4
Fruits	0.9	2.2	2.0	1.0	2.0	1.0	1.7	1.3	1.2	1.7	2.3	0.8
Fruit juices	0.8	0.4	0.9	0.4	0.9	0.4	0.9	0.4	0.7	0.6	0.8	0.4
Sugar-sweetened beverages	0.7	0.1	0.7	0.2	0.6	0.2	0.8	0.1	0.9	0.1	0.3	0.3
Sweets and desserts	1.5	0.9	1.3	0.9	1.4	0.9	1.1	1.2	1.3	0.9	1.0	1.1

Note: Analysis was conducted in the combined dataset.
*Starchy vegetables include potatoes and legumes.
Abbreviations: AHEI: Alternative Healthy Eating Index-2010; TLCDS: total low-carbohydrate diet score; ALCDS: animal low-carbohydrate diet score; VLCDS: vegetable low-carbohydrate diet score; HLCDS: healthy low-carbohydrate diet score; ULCDS: unhealthy low-carbohydrate diet score; Q1, Q5: Quintile 1, Quintile 5 of the specified dietary pattern.

© 2023 Liu B et al. *JAMA Network Open.*

eTable 5. Comparison between Independent correlation matrix vs. unstructured correlation matrix.

TLCDS		**Q1**	**Q2**	**Q3 (ref)**	**Q4**	**Q5**	**per-SD**	**p-values**
Corr=UN	Age adjusted	-0.17 (-0.21, -0.12)	-0.03 (-0.07, 0.02)	0	0.08 (0.03, 0.12)	-0.02 (-0.06, 0.03)	0.05 (0.04, 0.07)	<0.0001
	Multivariable adjusted	-0.20 (-0.25, -0.15)	-0.04 (-0.09, -0.00)	0	0.08 (0.03, 0.12)	-0.03 (-0.07, 0.02)	0.06 (0.04, 0.08)	<0.0001
Corr=IND	Age adjusted	-0.25 (-0.30, -0.20)	-0.05 (-0.09, -0.01)	0	0.04 (-0.00, 0.09)	-0.11 (-0.15, -0.06)	0.05 (0.03, 0.07)	<0.0001
	Multivariable adjusted	-0.29 (-0.34, -0.24)	-0.07 (-0.11, -0.02)	0	0.05 (0.00, 0.09)	-0.11 (-0.16, -0.06)	0.06 (0.04, 0.08)	<0.0001
ALCDS								
Corr=UN	Age adjusted	-0.20 (-0.25, -0.16)	-0.01 (-0.06, 0.03)	0	0.10 (0.06, 0.15)	0.11 (0.07, 0.16)	0.12 (0.10, 0.13)	<0.0001
	Multivariable adjusted	-0.23 (-0.28, -0.18)	-0.03 (-0.08, 0.01)	0	0.10 (0.05, 0.14)	0.11 (0.06, 0.16)	0.13 (0.11, 0.14)	<0.0001
Corr=IND	Age adjusted	-0.27 (-0.32, -0.22)	-0.04 (-0.08, 0.01)	0	0.05 (0.01, 0.10)	0.01 (-0.04, 0.06)	0.10 (0.08, 0.12)	<0.0001
	Multivariable adjusted	-0.30 (-0.35, -0.25)	-0.06 (-0.10, -0.01)	0	0.05 (-0.00, 0.09)	0.00 (-0.05, 0.05)	0.11 (0.09, 0.13)	<0.0001
VLCDS								
Corr=UN	Age adjusted	-0.15 (-0.20, -0.11)	-0.07 (-0.11, -0.03)	0	-0.05 (-0.09, -0.01)	-0.19 (-0.23, -0.15)	-0.02 (-0.04, -0.01)	0.01
	Multivariable adjusted	-0.17 (-0.22, -0.12)	-0.06 (-0.11, -0.02)	0	-0.05 (-0.10, -0.01)	-0.21 (-0.26, -0.17)	-0.03 (-0.04, -0.01)	0.004
Corr=IND	Age adjusted	-0.22 (-0.27, -0.17)	-0.10 (-0.14, -0.05)	0	-0.05 (-0.09, -0.00)	-0.21 (-0.26, -0.16)	-0.00 (-0.02, 0.01)	0.63
	Multivariable adjusted	-0.24 (-0.29, -0.19)	-0.09 (-0.14, -0.04)	0	-0.05 (-0.09, 0.00)	-0.23 (-0.28, -0.19)	-0.01 (-0.02, 0.01)	0.51
HLCDS								
Corr=UN	Age adjusted	0.33 (0.28, 0.37)	0.15 (0.11, 0.20)	0	-0.19 (-0.23, -0.15)	-0.62 (-0.66, -0.57)	-0.36 (-0.37, -0.34)	<0.0001
	Multivariable adjusted	0.32 (0.28, 0.37)	0.16 (0.12, 0.20)	0	-0.19 (-0.24, -0.15)	-0.64 (-0.69, -0.60)	-0.36 (-0.38, -0.35)	<0.0001
Corr=IND	Age adjusted	0.27 (0.22, 0.31)	0.13 (0.09, 0.18)	0	-0.19 (-0.24, -0.15)	-0.64 (-0.68, -0.59)	-0.33 (-0.35, -0.32)	<0.0001
	Multivariable adjusted	0.26 (0.22, 0.31)	0.14 (0.09, 0.18)	0	-0.19 (-0.24, -0.15)	-0.66 (-0.71, -0.61)	-0.34 (-0.36, -0.32)	<0.0001
ULCDS								
Corr=UN	Age adjusted	-0.56 (-0.60, -0.51)	-0.12 (-0.16, -0.07)	0	0.24 (0.20, 0.28)	0.42 (0.37, 0.46)	0.37 (0.35, 0.38)	<0.0001
	Multivariable adjusted	-0.60 (-0.65, -0.55)	-0.15 (-0.20, -0.11)	0	0.23 (0.19, 0.28)	0.42 (0.38, 0.47)	0.39 (0.37, 0.40)	<0.0001
Corr=IND	Age adjusted	-0.62 (-0.66, -0.57)	-0.13 (-0.17, -0.08)	0	0.20 (0.15, 0.24)	0.32 (0.27, 0.37)	0.34 (0.32, 0.36)	<0.0001
	Multivariable adjusted	-0.66 (-0.71, -0.61)	-0.16 (-0.20, -0.11)	0	0.19 (0.15, 0.24)	0.32 (0.28, 0.37)	0.36 (0.34, 0.38)	<0.0001

Note: Values are weight change in kilograms (95% CI). Q1 (Quintile 1): Large decrease; Q2 (Quintile 2): Moderate decrease; Q3 (Quintile 3): No change (referenced); Q4 (Quintile 4): Moderate increase; Q5 (Quintile 5): Large increase in low carbohydrate scores; p-values are for β for per-SD increase effect.

Multivariable generalized linear regression models (with unstructured OR independent correlation matrix and robust variance) were used. All analyses were run in the pooled dataset. Multivariable adjusted models adjusted for races and ethnicities (African American, Asian, White, Hispanic and Other, or missing), family history of diabetes (yes/no), baseline hypertension (yes/no), baseline hypercholesterolemia (yes/no), baseline total caloric intake (continuous, kcal/day), baseline body mass index (<25.0, 25.0-29.9, or ≥30.0 kg/m^2), change in smoking status (stayed never smoker, stayed former smoker, stayed current smoker, change from former to current smoker, change from never to current smoker, and change from current to former smoker), baseline and change in physical activity (METs-hour/week), change in alcohol consumption (continuous, g/day), postmenopausal hormone use (women only; premenopausal, never, former, current, or missing), oral contraceptive use (NHSII only; yes, no), and cohort (NHS, NHSII, HPFS).
Abbreviations: TLCDS: total low-carbohydrate diet score; ALCDS: animal low-carbohydrate diet score; VLCDS: vegetable low-carbohydrate diet score; HLCDS: healthy low-carbohydrate diet score ULCDS: unhealthy low-carbohydrate diet score; Corr=UN: unstructured correlation matrix; Corr=IND: independent correlation matrix.

© 2023 Liu B et al. *JAMA Network Open.*
