# Supplementary material for: Low-Carbohydrate Diet Macronutrient Quality and Weight Change
Source: JAMA Netw Open. 2023 Dec 27;6(12):e2349552. doi: 10.1001/jamanetworkopen.2023.49552 (PMC10753393; doi:10.1001/jamanetworkopen.2023.49552)
Supplement: Supplement 2. — Data Sharing Statement [file jamanetwopen-e2349552-s002.pdf]

Data Sharing Statement

Liu. Low-Carbohydrate Diet Macronutrient Quality and Weight Change. *JAMA Netw Open*. Published December 27, 2023. doi:10.1001/jamanetworkopen.2023.49552

Data
Data available: No
